# Transcription factors modulate RNA polymerase conformational equilibrium

**DOI:** 10.1038/s41467-022-29148-0

**Published:** 2022-03-22

**Authors:** Chengjin Zhu, Xieyang Guo, Philippe Dumas, Maria Takacs, Mo’men Abdelkareem, Arnaud Vanden Broeck, Charlotte Saint-André, Gabor Papai, Corinne Crucifix, Julio Ortiz, Albert Weixlbaumer

**Affiliations:** 1 Department of Integrated Structural Biology, Institut de Génétique et de Biologie Moléculaire et Cellulaire (IGBMC), 67404 Illkirch, France; 2 Université de Strasbourg, 67404 Illkirch, France; 3 Centre National de la Recherche Scientifique, UMR7104, 67404 Illkirch, France; 4 Institut National de la Santé et de la Recherche Médicale, U1258, 67404 Illkirch, France; 5 Present Address: GlaxoSmithKline, Gunnels Wood Road, Stevenage, Herts SG1 2NY UK; 6 Present Address: Forschungszentrum Jülich, Ernst Ruska-Centre for Microscopy and Spectroscopy with Electrons, Jülich, Germany

**Keywords:** Cryoelectron microscopy, Enzyme mechanisms, Transcriptional regulatory elements

## Abstract

RNA polymerase (RNAP) frequently pauses during the transcription of DNA to RNA to regulate gene expression. Transcription factors NusA and NusG modulate pausing, have opposing roles, but can bind RNAP simultaneously. Here we report cryo-EM reconstructions of *Escherichia coli* RNAP bound to NusG, or NusA, or both. RNAP conformational changes, referred to as swivelling, correlate with transcriptional pausing. NusA facilitates RNAP swivelling to further increase pausing, while NusG counteracts this role. Their structural effects are consistent with biochemical results on two categories of transcriptional pauses. In addition, the structures suggest a cooperative mechanism of NusA and NusG during Rho-mediated transcription termination. Our results provide a structural rationale for the stochastic nature of pausing and termination and how NusA and NusG can modulate it.

## Abstract (web summary)

Pausing of RNA polymerase (RNAP) and transcription is regulated by the NusA and NusG transcription factors in bacteria. Here the authors provide structural evidence for how they interact with RNAP to carry out their pausing roles and also reveal functions for NusA and NusG in transcription termination.

## Introduction

In the first step of gene expression, RNA polymerase (RNAP) transcribes DNA to RNA. RNAP is processive but pauses frequently. Pausing regulates gene expression and is also required to increase transcriptional fidelity and to terminate transcription in all kingdoms of life^1,2^. Regulatory pause signals are generally encoded in the DNA sequence^3,4^ and pauses are further modulated by a plethora of transcription factors (TFs)^5^. For example, NusA and NusG are essential TFs in bacteria. NusA enhances pauses stabilised by RNA hairpins^6,7^. In contrast, NusG, the only universally conserved TF, favours forward translocation and disfavours RNAP backtracking (RNAP reverse translocation along the DNA template) in Escherichia coli (*E. coli*)^8–10^. Both factors are involved in Rho-dependent transcription termination^11,12^ but their precise role is still under debate. NusA has been suggested to stimulate^13^ or inhibit Rho^14^. *E. coli* NusG consists of two domains. The N-terminal domain (NGN) binds RNAP and is connected through a flexible linker to the C-terminal KOW domain (NusG-KOW). NusG-KOW is thought to stimulate termination by activating termination factor Rho^15,16^ - at least for a subset of genes^17^. Quantitative mass spectrometry suggests that NusA and NusG are more abundant than RNAP in vivo^18^. Genome-wide studies indicated that NusA and NusG bind RNAP after initiation of transcription^19^. However, it is unclear if and how the two TFs regulate RNAP at the same time. Furthermore, structural information is available on the role of NusA and NusG in paused RNAP elongation complexes (EC)^7,10^, RNAP anti-termination complexes^20^ and ECs, which are intermediates leading to Rho-mediated transcription termination^11,12^. However, we lack an understanding of their role during canonical elongation. To address this question, we used single-particle electron cryo-microscopy (cryo-EM) to obtain reconstructions of three different RNAP ECs bound to NusG, NusA, or both and confirmed their role in biochemical assays. Here we show that NusG and NusA affect the RNAP conformational equilibrium, can cooperate and assist Rho-dependent termination, and they oppose each other’s effects in modulating transcriptional pausing.

## Results

### Structure and dynamics of the NusG-EC

**Fig. 1:**
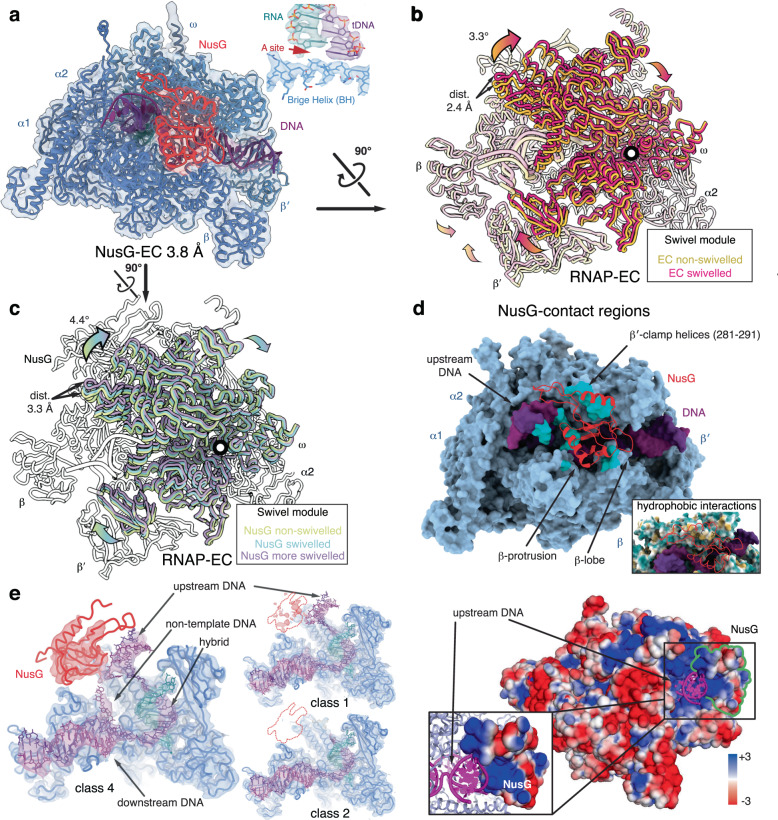
Structures of the NusG elongation complex (NusG-EC). **a** Consensus reconstruction of NusG-EC at 3.8 Å resolution and refined atomic model (left) shows the active site in a canonical post-translocated state (inset). NusG is coloured red, RNAP blue, DNA purple and RNA green. **b**, **c** 3D-classification resulted in 5 classes. **b** Classes 1 and 2 lack NusG (yellow and peach). The swivel module of these RNAP-ECs rotates up to 3.3° in comparison to the reference (PDB ID 6RH3). Comparing RNAP-EC class 1 and class 2, we observe 2.4 Å displacements furthest from the rotation axis. **c** Structural superpositions based on RNAP core module of classes 3 to 5 (light-green, cyan and purple), which contain strong NusG density and differ in their swivel module orientation. The swivel module rotates up to 4.4° (class 5) in comparison to the reference (PDB ID 6RH3, only one core module is shown for clarity in white). The swivel module rotations within classes 3 to 5 result in maximum displacements of 3.3 Å. **d** NGN (red) interacts with indicated regions of RNAP (turquoise) and provides a positively charged surface (bottom panel) that stabilises the upstream DNA (purple). **e** NusG (red) stabilises the upstream DNA duplex and the non-template DNA in the transcription bubble. Electron density of non-template DNA for NusG-containing class 4 (left) and classes lacking NusG (right).

We reconstituted an RNAP EC bound to NusG (NusG-EC) on a nucleic acid scaffold that directs the formation of a canonical post-translocated state and does not induce pausing (Supplementary Fig. 1a). The initial cryo-EM reconstruction we obtained resembled a recent cryo-EM structure of RNAP reconstituted at the *E. coli ops* pause bound to NusG and the phage λ N-dependent transcription anti-termination complex (λN-TAC)^10,20^ (Fig. 1a and Supplementary Fig. 1b–d).

The clamp and shelf module of RNAP (collectively called the swivel module, Supplementary Table 1) rotates relative to the RNAP core module producing interchangeable conformational states that have been termed non-swivelled and swivelled (Supplementary Table 2). The former is believed to be a requirement for substrate binding and catalysis by *E. coli* RNAP^21^, while the latter was observed in paused RNAP^7,21^.

The initial reconstruction of the NusG-EC resembled a non-swivelled conformation, but to further investigate the dynamics we performed heterogeneous refinement in cryoSPARC^22^. Particles were initially sorted into five classes (Supplementary Fig. 1d and Supplementary Table 3). Two classes (~40% of particles) contained very weak or no density for NusG but differed in their swivel module conformation (~1.3° to 3.4° rotation) (Fig. 1b and Supplementary Fig. 2a). The first, RNAP-EC class 1, resembles RNAP with a bound substrate prior to catalysis^23^. A substrate-bound RNAP trapped just before catalysis is likely conformationally homogenous and most representative for the active state. We performed all structural superpositions based on the RNAP core module and use the substrate-bound RNAP-EC (PDB ID 6RH3) as our reference (0° swivel rotation) in describing the swivel module rotation in the other states. These two classes represent a canonical RNAP EC without any bound TFs (RNAP-EC) and indicate the conformational freedom intrinsic to the swivel module of core RNAP bound to nucleic acids.

Three classes contain strong density for NusG, and we observe differences in mobile RNAP domains compared to previous reconstructions highlighting the flexibility of these surface-exposed modules (lineage-specific insertions SI1, SI2 and SI3) (Supplementary Fig. 2b and Supplementary Table 1). The three classes differ in the extent of swivelling (up to 4.4° rotation, Fig. 1c and Supplementary Table 2), and represent the conformational states of the NusG-EC. Similar results were independently obtained using 3D-classification in Relion^24^ as well as 3D-variability analysis in cryoSPARC^22^. Thus, the binding of NusG does not substantially restrict the swivel movement of RNAP.

As seen in previous NusG-containing complexes^10,11,20^, the NGN domain of NusG interacts with the β′ clamp helices (residues 281–291) (Fig. 1d), contacts the sugar phosphate backbone of the upstream DNA through a positively charged surface (Fig. 1d, bottom), and binds the β-protrusion and the β-gate loop within the RNAP β-lobe^25^ (Fig. 1d and Supplementary Table 1). The local resolution of these regions allowed most of these interfaces to be modelled confidently.

In the NusG-EC, the upstream DNA duplex and the single-stranded non-template DNA (ntDNA) in the transcription bubble were more ordered compared to the RNAP-EC (Fig. 1e). The presence of NusG extends the positively charged surface area of RNAP in contact with the negatively charged upstream DNA duplex and single-stranded ntDNA (Fig. 1d, bottom). Thus, like in an EC reconstituted at a pause site^10^, NusG guides and restrains the movement of the single-stranded ntDNA and stabilises the upstream DNA duplex in a canonical EC. Stabilisation of base-pairing within the upstream DNA duplex, which reanneals at the upstream fork, might favour forward translocation and prevent backtracking as suggested previously^9,10^. A similar proposal has been made for DNA stabilisation by phage λ protein N via a positively charged domain that thereby favours RNAP processivity^20^.

The binding of NGN to RNAP was previously suggested to be incompatible with swivelling, due to steric clashes^10^. However, our reconstructions show that the swivel module can rotate 4.4° in presence of NusG because the tip of the β-protrusion is flexible and accommodates NusG during swivelling (Supplementary Fig. 2c and Movie 1). NusG does not appear to alter the intrinsic capability of RNAP to swivel, but several additional contacts with the β-lobe, β-protrusion and β-clamp may disfavour more extensive swivelling (Supplementary Fig. 2c, bottom right).

Previous studies did not observe NusG-KOW, and this was attributed to the flexibility of the intervening linker. We observed, however, that upon low-pass-filtering to 10 Å, the full-length NusG and the β-flap tip helix (FTH) of RNAP are visible in all classes containing NusG (Supplementary Fig. 2d). NusG-KOW was visible as extra density on top of the FTH. Together with the NGN domain it arcs around the upstream DNA, which may further limit its mobility and stabilise it (Supplementary Fig. 2e). A paralogue of NusG, the TF RfaH, also binds the FTH through its C-terminal domain in a similar manner^10^.

### Structure and dynamics of the NusA-EC

**Fig. 2:**
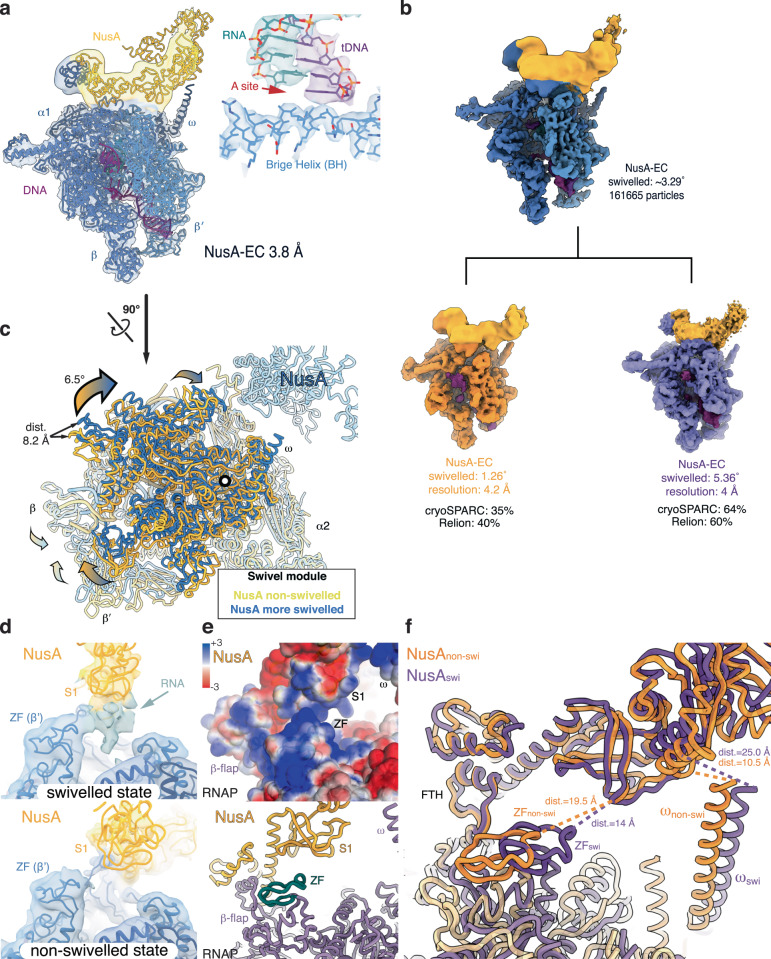
Structures of the NusA elongation complex (NusA-EC). **a** Consensus reconstruction of NusA-EC at 3.8 Å resolution shows NusA (yellow) bound to RNAP (blue) that is in a post-translocated state (inset). The map showing NusA was low-pass filtered to 10 Å. **b** 3D-classification of NusA-EC. The refined structure with all particles (shown in blue on the top) reached the highest nominal resolution (3.8 Å). Approximately one third of particles were assigned to the non-swivelled conformation and the other two thirds were in a swivelled conformation. **c** 3D-variability analysis of the NusA-EC showed extensive rotation of the swivel module (6.5˚) relative to the non-swivelled reference (substrate-bound RNAP EC, PDB ID 6RH3), resulting in over 8.2 Å displacements at the tips of the swivel module. **d** EM density for the nascent RNA (green) and approaching NusA S1 domain (yellow) to the RNAP zinc finger (ZF) (blue) was observed in the swivelled state but not the non-swivelled state of NusA-EC (models were aligned based on the swivel module). **e** Electrostatic surface (top) and structural model (cartoon, bottom) of the swivelled state shows that S1 and ZF are both positively charged, and are brought together by their interaction with the nascent transcript (see electron density in **d**). **f** Comparison of the swivelled (purple) and non-swivelled (orange) NusA-ECs shows interactions between RNAP and NusA that stabilise the swivelled state. This interaction may contribute to the ability of NusA to shift the conformational equilibrium towards a swivelled state by anchoring on the flap-tip-helix (FTH) and indirectly contacting the β’-ZF. The distance between ω (R91) and NusA (G268) and the distance between β’-ZF (L78) and NusA (G176) are indicated.

To compare the effect of NusG on an RNAP-EC with NusA, which stimulates pausing, we reconstituted a complex using the same nucleic acid scaffold in presence of NusA (Supplementary Fig. 1a). A reconstruction of the NusA-EC was obtained, which was refined to a nominal resolution of 3.8 Å (Fig. 2a and Supplementary Fig. 3a). The local resolution for NusA was lower, reflecting its intrinsic flexibility and movement relative to RNAP, which is consistent with earlier observations (Supplementary Fig. 3b–c)^7,20^. The consensus reconstruction closely resembles a previous structure of a NusA-RNAP complex in an RNA hairpin stabilised paused state (backbone RMSD for RNAP 1.3 Å, Supplementary Fig. 3d)^7^. NusA is positioned close to the RNA exit channel, binds the FTH, the C-terminal domains of the RNAP α-subunits (α-CTDs) and the C-terminal tip of the ω-subunit. However, in contrast to the paused state, RNAP is post-translocated with an active site compatible with substrate binding (Fig. 2a). In the consensus refinement of the NusA-EC, the swivel module is more swivelled compared to NusG-EC (3.3° rotation relative to the substrate-bound RNAP EC, PDB ID 6RH3, Supplementary Table 2). Heterogenous refinements in cryoSPARC produced two classes: one class produced by approximately two-thirds of the particle is in a swivelled but post-translocated state, while one third is in a non-swivelled post-translocated state (Fig. 2b and Supplementary Tables 2 and 4). Similar results were independently obtained using 3D-classification in Relion^24^. To further model the conformational dynamics, we employed 3D-variability analysis in cryoSPARC. The RNAP swivel module adopts a continuum of positions with the most extreme swivelled position rotated by 6.5˚ relative to the non-swivelled state (Fig. 2c and Movie 2).

NusA and RNAP provide a positively charged path for the nascent RNA guiding it to the RNA binding domains of NusA (S1, KH1 and KH2)^7,26,27^. 3D-variability analysis allowed us to estimate the rotational range of NusA relative to RNAP (Supplementary Fig. 3c), and of RNAP swivelling. NusA maintains contacts via the NusA N-terminal domain (NusA-NTD) and the RNAP FTH, the NusA-NTD and the RNAP α1-CTD, and likely the NusA-AR2 domain and RNAP α2-CTD as observed before^7^. The NusA KH domains contact the RNAP ω-subunit, but this interaction is dynamic and not stable (Supplementary Fig. 3e). The S1 domain of NusA approaches the RNAP β′-zinc finger (ZF) in the swivelled state. This restricts the space available to the nascent RNA, which consequently shows more ordered density in comparison to the non-swivelled state (Fig. 2d). The nascent RNA likely stabilises the closely positioned RNAP ZF and S1 domain, which would otherwise be repelled by their positively charged surfaces (Fig. 2e). This mutual contact with the nascent RNA may stabilise the swivelled state and thus allow NusA to facilitate more extensive swivelling (Fig. 2f). The nascent RNA in our reconstructions is 14 nucleotides long. Longer or more structured RNA transcripts may modulate these effects as seen in anti-termination complexes^20^.

The NusA-EC exhibits more pronounced swivelling compared to NusG-EC or RNAP lacking bound TFs (Supplementary Fig. 4a and Supplementary Table 2). Extensive RNAP swivelling is thus not restricted to complexes in stabilized, paused states such as the *his-*pause and can also be accessed by a post-translocated RNAP. Importantly, increased occupation of a swivelled state in ECs bound by NusA likely increases the probability of pausing when encountering a DNA encoded pause signal.

### Structure and dynamics of the NusA-NusG-EC

**Fig. 3:**
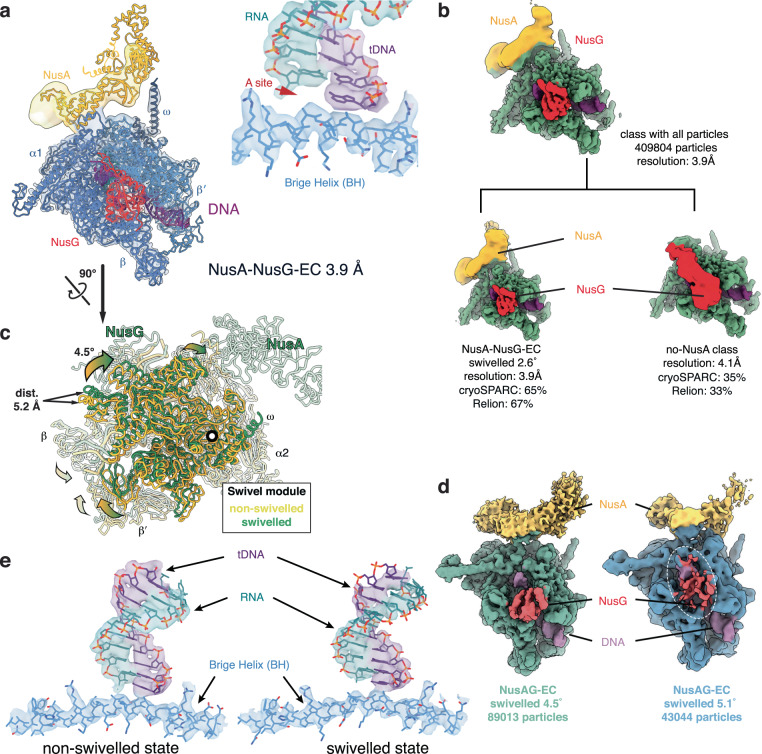
Structures of the NusA-NusG-EC. **a** The NusA-NusG-EC reconstruction refined to an overall resolution of 3.9 Å. The refined atomic model shows a post-translocated active site (inset). NusA is shown in yellow, NusG in red. **b** 3D-classification of all particles resulted in 2 classes: one contains both factors (NusA-NusG-EC), the other lacks NusA. The NusA-NusG-EC class refined to 3.9 Å (shown in panel a). However, the NusG-KOW EM density is not resolved. The class lacking NusA refined to 4.1 Å. Low-pass filtering the NusG density to 10 Å resolution (red, bottom right), reveals the contact between NusG-KOW and FTH. **c** The swivel module can rotate up to 4.5° and sample a range of orientations intermediate to NusG-EC and NusA-EC. Displacements furthest away from the rotation axis are up to 5.2 Å. **d** NusG and NusA bind to RNAP at swivel angles up to at least 4.5°, but EM density for NusG was weaker at more extreme swivel angles (~5° - seen in 3D variability analysis), while that for NusA (yellow) was not. **e** EM density for the RNA-DNA hybrid (indicated by short arrows) was better defined in non-swivelled states, suggesting a more constrained RNA substrate. Here, swivelled NusA-EC, and non-swivelled NusA-NusG-EC classes, which refined to the same resolution are compared at the same contour level.

Despite their opposing roles on pausing, NusA and NusG bind RNAP simultaneously. To investigate this, we reconstituted an RNAP EC in presence of both factors on the same nucleic acid scaffold used for the complexes described thus far (NusA-NusG-EC) (Supplementary Fig. 1a). 3D-classification of all particles used for cryo-EM reconstructions revealed two major subsets: the first contained both factors bound to RNAP (65% of particles, nominal resolution 3.9 Å, the consensus NusA-NusG-EC) (Fig. 3a and Supplementary Fig. 4b). The second corresponded to NusG-EC (35% of particles, nominal resolution 4.1 Å, Fig. 3b and Supplementary Table 5).

In the NusA-NusG-EC, both factors bind their expected sites on RNAP, in agreement with the structures of RNAP bound to the individual proteins. However, in contrast to the NusG-EC that lacked NusA, the FTH is occupied by NusA-NTD rather than the NusG-KOW domain. Low-pass filtering the maps for NusA-NusG-EC did not reveal the location of the NusG-KOW suggesting it is now highly flexible. Thus, NusA and NusG appear to compete for binding the FTH. Further 3D-classification revealed swivelling movement of RNAP as observed in NusG-EC and NusA-EC. RNAP swivels up to an angle of 4.5° (Fig. 3c and Supplementary Table 2) with both factors present, thus the RNAP swivelling range appears to be at an intermediate level between NusA-EC and NusG-EC, which suggests the two factors oppose each other’s effect. However, density for NusG was weaker in a small subpopulation of RNAP that swivelled up to 5.1° suggesting partial dissociation of NusG (Fig. 3d). Interestingly, density for the RNA-DNA hybrid appeared stronger and better-defined in the non-swivelled than the swivelled conformation (Fig. 3e). This is consistent with the proposal that non-swivelled RNAP is prone to catalysis^21^ and stabilises the RNA-DNA hybrid.

### NusA-stimulated pausing at an RNA hairpin is counteracted by NusG

**Fig. 4:**
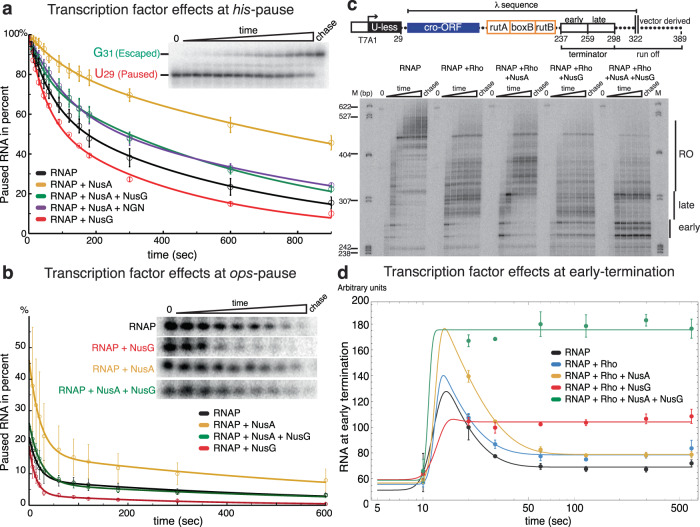
Transcription elongation and termination kinetics regulated by NusA and NusG. For all panels, RNA was quantified in triplicates (*n* = 3) and error bars indicate standard deviations from the mean (the source data are provided as a Source Data file). **a** RNAP transcription elongation complexes were assembled upstream of the *E. coli his*-pause to measure pause escape rates. Pause escape rates quantified from denaturing gel electrophoretic assays (representative result shown in inset). NusA decreased pause escape rates 2-fold (yellow). NusG slightly increased escape rates (red). When both factors were present (or NusA and NGN), rates were almost reaching the level of RNAP alone (green, purple and black). **b** Transcriptional pausing at the *ops*-pause measured in single-round transcription assays and analysed by denaturing gel electrophoresis (inset). NusG (red) increased rates of escape from the pause, while NusA (yellow) increased the fraction of paused RNAP and slowed down escape rates. When both factors were present, they cancelled each other’s effect (black and green). **c** Schematic of the assay template DNA (top), and a representative gel of the Rho-dependent termination assay in the presence or absence of TFs (bottom). The template DNA contained a T7A1 promoter, a U-less region ending with A29, the phage λ *cro* gene and λtR1 terminator with the binding site for Rho (rutA, boxB and rutB). Transcription terminated in the early or the late region (early: 237-259, late: 259-298). 298-322 represents the sequence downstream of the λ cro-tR1, and 322-389 is derived from the vector. The terminator and the run off region are indicated. Terminated and run-off transcription products were measured over time in single-round transcription assays. In the absence of TFs, most RNA products by-passed terminators and transcription ended in the run-off region. Rho caused termination in the late region. NusA increased pausing in the terminator regions but RNAP eventually extended those transcripts. Termination occurred efficiently in presence of NusG and even more so in the early region when both NusG and NusA were present. **d** Quantification of the early termination efficiency in presence of the indicated set of transcription factors over time. Plotted curves were obtained by mathematical modelling (see Methods section for details), y axis represents arbitrary units.

We sought to study the effects of NusG and NusA on transcription elongation, both individually or in combination and performed RNA extension assays at two different regulatory pause sites. First, we reconstituted RNAP-ECs at the *E. coli his*-pause, a well-characterised pause involved in attenuation in the histidine biosynthesis operon and stabilized by an RNA hairpin^28^ (Supplementary Fig. 5a). We measured pause-escape rates (see Methods section for details, Supplementary Fig. 5b). Under saturating concentrations of TF, NusA decreased pause escape rates ~2-fold compared to RNAP alone (Supplementary Fig. 5b), consistent with previous reports^6,29,30^. In the presence of NusG, the pause duration is slightly shorter than for RNAP alone, resembling results obtained with the NusG paralogue RfaH^25^ (Fig. 4a, compare red and black curve). When both factors were included in the reaction, pausing time was shorter than with NusA alone, but longer than RNAP without either TF (Fig. 4a, compare green curve with yellow and black curve). This result is consistent with our structural data, and suggests the effect of NusA in stabilising the paused state is partially counteracted by NusG. To clarify if the effect of NusG in reducing pausing relied on its ability to compete with NusA for binding to the FTH domain of RNAP, we repeated the assay with a truncated NusG variant containing only the NGN domain (Supplementary Fig. 5c). NGN had the same effect as the full-length NusG and increased pause-escape rates from the *his*-pause in presence of NusA. We conclude that the NGN domain alone is sufficient to reduce the effect of NusA (Fig. 4a) and NusG therefore counteracts NusA through changes to RNAP swivelling equilibrium rather than competing for binding to the RNAP FTH domain.

### NusA offsets NusG in suppressing backtrack-pausing at the *ops*-pause

To compare a hairpin-mediated pause to a backtrack stabilized pause (see Methods section for details), we tested the effects of NusA and NusG on the *ops-*pause^6^ (Supplementary Fig. 5d). Addition of NusG decreased the fraction of paused RNAP and pause duration at *ops*, confirming earlier results^6^ (Fig. 4b and Supplementary Fig. 5e–g). By contrast, addition of NusA reproducibly increased the fraction of paused RNAP and extended pause duration (Fig. 4b). When both factors are present in the reaction, they cancel each other’s effect and pausing returns to levels seen in absence of any TFs (Fig. 4b). Thus, similar to a hairpin-mediated pause such as the *his*-pause, NusA and NusG have opposing effects on the *ops*-pause.

### NusA and NusG cooperate in Rho-mediated termination of transcription

Our structural results suggested the NusG-KOW domain is more accessible to bind other TF when NusA occupies the FTH. NusG-KOW stimulates termination factor Rho^16^. We therefore hypothesised that NusA may indirectly favour termination by Rho both through the release of NusG-KOW from the FTH and by increasing the probability of pausing. To test if NusG and NusA can cooperate during Rho-dependent termination, we carried out single-round transcription assays and monitored termination in presence of purified Rho at the paradigmatic λ tR1 terminator (Fig. 4c). This terminator has previously been characterised to have three (called I, II and III) or two (early and late) regions where termination occurs^16,31^. Measuring the cumulative termination at these sites, we observed that only approximately 13% of transcripts terminate within the early and late regions in absence of Rho, but this increased to approximately 45% in the presence of Rho (Supplementary Fig. 6a). Addition of NusA with Rho reduced termination efficiency to 23%, consistent with earlier reports^14^, while addition of NusG and Rho increased termination slightly to 51%. In presence of NusA and NusG termination efficiency was 56%. Although this may suggest cooperativity between NusA and NusG in Rho-mediated termination of transcription, the experimental errors limit our ability to draw strong conclusions.

Analysis for individual termination regions separately provides a more detailed picture. In the early region, Rho alone or Rho with NusA did not increase termination efficiency. In contrast, Rho in combination with NusG increased termination efficiency approximately 3-fold, consistent with recent data^11,12,14,16^. When both NusA and NusG were present, termination efficiency increased 6-fold compared to RNAP with Rho alone (Supplementary Fig. 6b and Fig. 4c). In the late region, addition of Rho increased termination efficiency four-fold. NusA shifted termination further downstream and consequently reduced termination efficiency in the late region two-fold, similar to previous observations^11,12^. Addition of NusG alone had no strong effect. However, NusG and NusA together, while not increasing termination efficiency, appeared to focus termination to a narrower region (Fig. 4c and Supplementary Fig. 6b).

We further analysed transcription termination over time at the early region, where termination occurs in a relatively narrow window (Fig. 4c). We assume a kinetic partitioning into three populations: (i) RNAP ignores the terminator, does not pause and only contributes a negligible amount of signal; (ii) RNAP pauses but eventually bypasses and resumes transcription; or (iii) RNAP pauses and terminates so RNA products accumulate. We used a simple kinetic model, which does not take the first but only the latter two populations into account (Supplementary Fig. 6c). Using this model, we interpret our observations in the following way: a fraction of RNAP briefly pauses within the early termination region and Rho, if present, has a modest effect in increasing termination efficiency (Fig. 4c, d, compare black curve with blue curve, Supplementary Fig. 6d, compare values for probability of terminating transcription P_off_). Addition of NusA increased the fraction of paused RNAP but the overall termination efficiency is comparable to Rho alone and the majority of paused RNAP resumes transcription (Fig. 4d, compare blue curve with yellow curve, Supplementary Fig. 6d, compare values for coefficient A, which reflects paused fraction of RNAP and P_off_). Addition of NusG increased termination efficiency and it appears every paused RNAP is now committed to terminate (Fig. 4d, compare blue and red curve, Supplementary Fig. 6d, compare coefficient A and termination probability P_off_). However, a cooperative effect occurs in presence of both NusA and NusG. While the fraction of paused RNAP may not reach the same level as in the presence of NusA alone, almost all of them terminate to increase the overall termination efficiency six-fold compared to RNAP with Rho (Fig. 4d, compare blue, red and green curve; Supplementary Fig. 6d, compare coefficient A and termination probability P_off_). Importantly, deletion of NusG-KOW abolished this effect in agreement with previous results^16^ (Supplementary Fig. 6e–f). We conclude that NusA and NusG can cooperate to increase the overall termination efficiency in the early region of the λ tR1 terminator.

## Discussion

Distinct RNAP conformations reflect specific states in the transcription cycle. RNAP swivelling has been observed during backtracking and transcriptional pausing, and was proposed to be prevented during anti-termination^7,20,21,23,32^. The measured extent of RNAP swivelling is consistent with these proposals (Supplementary Table 2). In contrast, a non-swivelled RNAP conformation had been proposed to be required for catalysis^21^. This is consistent with the observation that an entire ensemble of substrate bound complexes adopts a non-swivelled conformation^23^. Here we show swivelling also occurs in a canonical EC. Rather than enforcing a specific discrete RNAP conformation, NusG or NusA tune transcription by adjusting the range of accessible swivel module orientations. NusA favours transcriptional pauses that depend on RNAP adopting the swivelled state. We anticipate that NusA enhances intrinsic termination in the same manner. NusA may indirectly stabilise RNAP in the swivelled state through an interaction with the nascent RNA and the ZF. When the ribosome approaches RNAP in a physically coupled transcription-translation complex, NusA is no longer able to approach the ZF and so transcriptional pausing might be decreased^33^.

NusG disfavours pauses by promoting a non-swivelled state. As a consequence, NusA and NusG produce opposing effects during transcriptional pausing. NusG-KOW can interact with the RNAP FTH. When NusA binds RNAP concurrently with NusG, this contact is no longer possible because NusA occupies the FTH and NusG-KOW is consequently accessible to interact with other partners such as Rho. When RNAP in presence of Rho reaches the early termination region of the λ tR1 terminator, NusA increases pausing without increasing termination efficiency suggesting pausing alone is not sufficient. Instead, termination occurs further downstream. On the contrary, NusG increases termination efficiency for the paused fraction of RNAP in presence of Rho. When both NusA and NusG are present, pausing facilitated by NusA coupled with enhanced termination efficiency by NusG lead to an overall increase in termination efficiency. This is consistent with recent findings that NusG-KOW stimulates Rho ring closure on suboptimal RNA substrates^16^. NusA and NusG are therefore able to tune transcription elongation and termination cooperatively through their effects on the RNAP conformation.

## Methods

### Escherichia coli strains

TOP10 cells (Invitrogen) were used for plasmid construction. *E. coli* LACR II (Low Abundance of Cellular RNases), a derivative of *E. coli* LOBSTR^34^, was constructed and used for recombinant protein expression. LACR II is a double knock-out for the most abundant RNases (RNase I and RNase II, *rna*^*-*^ *rnb*^*-*^) to decrease the amount of RNase contamination in the purified protein sample (details to be published elsewhere). Similarly, a derivative of *E. coli* BL21(DE3), which was a generous gift from the Deutscher lab and is also a double knock-out for RNase I and RNase II (*rna*^*-*^ *rnb*^*-*^)^35^ was also used.

### Purification of RNAP

pVS11_rpoA_rpoB_rpoC_HRV3C_His_10__rpoZ vector containing the genes encoding the *E. coli* RNAP core enzyme subunits with a C-terminal His_10_-tag on the β’-subunit was transformed into *E. coli* LACR II. pACYC_Duet1_rpoZ was co-transformed to avoid sub-stoichiometric amounts of the RNAP ω subunit. *E. coli* RNAP was overexpressed in 6 L LB culture (100 μg/ml ampicillin, 34 μg/ml chloramphenicol), which was then induced by adding 0.5 mM IPTG at an OD_600_ of 0.6–0.8 and incubated at 18 °C overnight. After centrifugation (30 min, 4500×*g* at 4 °C), the cell pellet was resuspended in 5 volumes of lysis buffer (50 mM Tris-HCl pH 8.0, 5% glycerol, 1 mM EDTA pH 8.0, 10 mM DTT, 0.1 mM PMSF, 1 mM benzamidine, 10 μM ZnCl_2_, 100 mg DNase I, cOmplete EDTA-free protease inhibitor cocktail (Sigma-Aldrich, 1 tablet/50 ml)) followed by sonication. The insoluble material was removed by centrifugation (40,000×*g*, 30 min, 4 °C), and RNAP was isolated from the supernatant by polyethyleneimine (PEI) precipitation (0.6% final concentration). The PEI precipitate was washed twice with 100 mL PEI wash buffer (10 mM Tris-HCl pH 8.0, 5% glycerol, 0.1 mM EDTA pH 8.0, 0.5 M NaCl, 1 mM DTT, 0.1 mM PMSF, 1 mM benzamidine, 10 μM ZnCl_2_) and RNAP was extracted three times with 10-20 mL PEI elution buffer (10 mM Tris-HCl pH 8.0, 5% glycerol, 0.1 mM EDTA pH 8.0, 1 M NaCl, 1 mM DTT, 0.1 mM PMSF, 1 mM benzamidine, 10 μM ZnCl_2_), The PEI elution sample was precipitated by adding ammonium sulfate (~35 mg/(100 ml of sample)), as described previously^36^. The precipitate was collected by centrifugation (10,000×*g*, 10 min, 4 °C) and resuspended in IMAC buffer (20 mM Tris-HCl pH 8.0, 1 M NaCl, 5% glycerol, 5 mM β-mercaptoethanol, 0.1 mM PMSF, 1 mM benzamidine, 10 mM ZnCl_2_) and passed over a 20 mL Ni-IMAC Sepharose HP column (GE Healthcare) using a step gradient with 250 mM imidazole in IMAC buffer for the elution (IMAC buffer for 2 column volumes (CVs), 5 mM imidazole wash for 2 CVs, gradient from 5 to 40 mM imidazole over 1 CV, 40 mM imidazole for 5 CV, and final step to 250 mM Imidazole). In order to cleave the affinity tag, peak fractions were pooled and dialysed in the presence of HRV3C (PreScission) protease (1 mg HRV3C per 8 mg of protein) overnight in dialysis buffer (20 mM Tris-HCl pH 8.0, 1 M NaCl, 5% glycerol, 5 mM β-mercaptoethanol, 10 μM ZnCl_2_). The non-cleaved/cleaved RNAP, the His_10_-tag and HRV3C were separated by reloading the sample on the IMAC column. The cleaved RNAP was then dialysed into Bio-Rex buffer (10 mM Tris-HCl pH 8.0, 5% glycerol, 0.1 mM EDTA, 1 mM DTT, 0.1 mM PMSF, 1 mM benzamidine, 10 μM ZnCl_2_) until conductivity was ≤10 mS/cm. The RNAP was then loaded on a 50 mL Bio-Rex 70 column (BIO-RAD) and eluted using a linear gradient into Bio-Rex buffer with 1 M NaCl over 5 CVs. Afterwards, the concentrated peak was purified by gel filtration using a HiLoad Superdex 200 PG 26/600 column (GE Healthcare) equilibrated with GF buffer (10 mM HEPES pH 8.0, 0.5 M KCl, 1% glycerol, 2 mM DTT, 0.1 mM PMSF, 1 mM benzamidine, 10 μM ZnCl_2_, 1 mM MgCl_2_). In the end, the protein was dialysed into EM buffer (10 mM HEPES pH 8.0, 150 mM KOAc, 2 mM DTT, 10 μM ZnCl_2_, 5 mM Mg(OAc)_2_), concentrated to ~80 mg/ml, aliquoted, flash frozen in liquid nitrogen, and stored at −80 °C.

### Purification of NusG/NGN

pSKB2_His_6__ppx_EcoNusG_FL or pAX0_His_10__ppx_EcoNusG_NTD plasmids encode His6-tags at the N-terminus of NusG full length (NusG) or NusG N-terminal domain (NGN) followed by an HRV3C cleavage site. NusG coding plasmids (NusG/NGN) were transformed into *E. coli* LACRII (*rna*^*-*^ *rnb*^*-*^) strain. NGN and NusG were separately overexpressed in 6 L LB culture (50 μg/ml kanamycin), induced by 1 mM IPTG at an OD_600_ of 0.6 for 3 h at 37 °C. The cells were harvested by centrifugation (30 min 4500×*g* at 4 °C), resuspended in 4 volumes of lysis buffer (50 mM Tris-HCl pH 8.0, 2 mM EDTA, 233 mM NaCl, 5% glycerol, 5 mM β-mercaptoethanol, 0.1 mM PMSF, 1 mM benzamidine, cOmplete EDTA-free protease inhibitor cocktail (Sigma-Aldrich, 1 tablet/50 ml) and lysed by sonication (amplitude 60%, 2 min with pulse-on/off durations of 0.5 sec). The lysates were cleared by centrifugation at 40,000×*g* for 30 min at 4 °C in a 50.2 Ti rotor (Beckman Coulter). The nucleic acids and their interacting proteins were precipitated by adding 0.6% of polyethyleneimine and removed by centrifugation at 45,000×*g* for 20 min at 4 °C. Ammonium sulfate was added to the supernatant to a final concentration of 0.37 g/ml and the precipitant was collected by centrifugation at 45,000×*g* for 20 min at 4 °C. The pellet was then resuspended in IMAC buffer A (50 mM Tris-HCl pH 8.0, 500 mM NaCl, 5 mM imidazole, 1 mM β-mercaptoethanol, 0.1 mM PMSF, 1 mM benzamidine). NusG proteins were first purified by passing the supernatant over a 5 mL HiTrap IMAC HP column (GE Healthcare) charged with NiSO_4_ and eluted at 200 mM imidazole using a step gradient with buffer B (50 mM Tris-HCl pH 8.0, 500 mM NaCl, 500 mM imidazole, 1 mM β-mercaptoethanol, 0.1 mM PMSF, 1 mM benzamidine). The peak fraction was then dialysed overnight against dialysis buffer (50 mM Tris-HCl pH 8.0, 250 mM NaCl, 5% glycerol, 1 mM β-mercaptoethanol) in the presence of HRV3C (PreScission) protease (1 mg HRV3C per 18 mg of protein). The dialysed sample was reloaded on the IMAC column. Cleaved NusG proteins bind weakly to the IMAC column and were eluted at ~60 mM Imidazole, and further dialysed into ion-exchange buffer A (10 mM Tris-HCl pH 8.0, 0.1 mM EDTA, 5% glycerol, 1 mM DTT, 0.1 mM PMSF, 1 mM benzamidine). The proteins were then loaded on a 5 mL HiTrap Q HP column (GE Healthcare) and purified using a gradient of 0-100% ion-exchange buffer B (10 mM Tris-HCl pH 8.0, 200 mM NaCl, 0.1 mM EDTA, 5% glycerol, 1 mM DTT, 0.1 mM PMSF, 1 mM benzamidine) for 20 CVs to elute NusG. The peak was concentrated and further purified by gel filtration using a Superdex 75 16/600 column equilibrated with GF buffer (10 mM Tris-HCl pH 8.0, 500 mM NaCl, 0.1 mM EDTA, 5% glycerol, 1 mM DTT, 0.1 mM PMSF, 1 mM benzamidine). The final proteins were concentrated to 5 mg/ml, aliquots were flash frozen, and stored at −80 °C.

### Purification of NusA

*E. coli* NusA with a His_10_-tag at the N-terminus was overexpressed in *E. coli* BL21 (rna- rnb-) strain. In 6 L LB culture (50 mg/l kanamycin), expression was induced with 1 mM IPTG at an OD_600_ of 0.7 for 3 hours at 37 °C. Bacteria were pelleted (30 min 4500×*g* at 4 °C), resuspended in 5 volumes of lysis buffer (50 mM Tris-HCl pH 8.0, 0.5 M NaCl, 10 mM imidazole, 2 mM β-mercaptoethanol, 0.1 mM PMSF, 1 mM benzamidine, DNase I (0.5 mg/250 g cell), cOmplete EDTA-free protease inhibitor cocktail (Sigma-Aldrich, 1 tablet/50 ml) and lysed using sonication (amplitude 60%, 2 min at pulse on/off durations of 0.5 sec). The lysates were cleared by centrifugation at 40,000×*g* for 30 min and the supernatant was loaded on two 5 mL HiTrap IMAC HP columns (GE Healthcare) and eluted using a linear gradient into lysis buffer containing 250 mM imidazole in 10 CVs. His-tagged HRV3C (PreScission) protease was added to peak fractions containing NusA and dialysed overnight against lysis buffer with 50 mM NaCl. Cleaved NusA was separated from non-cleaved protein, and the His-tagged protease by reloading onto the IMAC column. The flow-through was applied to a 5 ml HiTrap Q HP column (GE Healthcare). NusA was eluted using a gradient over 10 CVs into lysis buffer plus 1 M NaCl. The peak was concentrated and further purified by gel filtration using a Superdex 75 16/60 column equilibrated with GF buffer (10 mM HEPES pH 8.0, 0.15 M NaCl, 0.1 mM EDTA, 1 mM DTT). The final protein was concentrated to >50 mg/ml, aliquots were flash frozen, and stored at −80 °C.

### Purification of *E. coli* transcription termination factor Rho

The pET28b plasmid containing the termination factor *rho* gene was transformed and overexpressed in *E. coli* BL21-DE3 (pLysS) strain. In 6 L LB culture (50 μg/ml kanamycin and 37 μg/ml chloramphenicol), expression was induced with 0.8 mM IPTG at OD_600_ of 0.6–0.8 for 3 hours at 37 °C. Bacteria were pelleted (30 min 4500×*g* at 4 °C), resuspended in 5 volumes of lysis buffer (50 mM Tris-HCl pH 7.5, 5% glycerol, 5 mM EDTA pH 8, 0.1 mM DTT, 100 mM NaCl, 10 mM imidazole, 1 mM β-mercaptoethanol, 1 mM PMSF, 1 mM benzamidine, cOmplete EDTA-free protease inhibitor cocktail (Sigma-Aldrich, 1 tablet/50 ml) and 0.01 volume of 5% sodium deoxycholate) and lysed using sonication (amplitude 60%, 2 min at pulse on/off durations of 0.5 sec). DNase I (0.5 mg/250 g cells) and 24 mM MgCl_2_ were added to the sample and incubated for 20 min on ice. The lysates were cleared by centrifugation at 30,000×*g* for 30 min at 4 °C (50.2 Ti rotor, Beckman Coulter). 5% v/v polyethyleneimine was added to the supernatant and the precipitate was removed by centrifugation at 30,000×*g* for 15 min at 4 °C. 0.5 g/mL of ammonium sulfate was added to the supernatant. After one hour on ice, the precipitate was collected by centrifugation at 30,000×*g* for 20 min at 4 °C. The pellet was resuspended in and dialysed overnight against buffer A (10 mM Tris-HCl pH 7.6, 5% glycerol, 0.1 mM EDTA pH 8, 0.1 mM DTT, 100 mM NaCl). Rho was first purified by passing the supernatant through a 20 mL SP sepharose column (GE Healthcare) using a 0-60% linear gradient of buffer B (10 mM Tris-HCl pH 7.6, 5% glycerol, 0.1 mM EDTA pH 8, 0.1 mM DTT, 1 M NaCl) over 11 CVs. The pooled-peak fractions were loaded on a 5 ml Heparin HP column after setting the salt concentration to 400 mM (by dilution if necessary). The column was equilibrated with Buffer A, and Rho was eluted using a 0-100% linear gradient into buffer B over 20 CVs. The peak was concentrated and further purified by gel filtration using a Superdex 200 16/60 column equilibrated with GF buffer (20 mM Tris-HCl pH 7.9, 5% glycerol, 0.2 mM EDTA pH 8, 500 mM KCl, 0.2 mM DTT). The peak fractions containing Rho were then dialysed against storage buffer (10 mM Tris-HCl pH 8, 50% glycerol, 0.1 mM EDTA pH 8, 150 mM KCl, 1 mM DTT), concentrated to 80 mg/ml, flash frozen and stored at −80 °C.

### DNA/RNA oligonucleotides

DNA (TriLink) and RNA (Dharmacon) oligonucleotides were chemically synthesised and gel purified by the manufacturer. RNA was deprotected following the protocols provided by the manufacturer. Both DNA and RNA were dissolved in RNase-free water and aliquots were stored at −80 °C.

### EMSA assay

Complex formation was confirmed by electro mobility shift assays (PhastGel system, GE Healthcare). The isolated nucleic acid scaffold was prepared in a volume of 100 μl by mixing 50 μM RNA, 100 μM template DNA (tDNA) and 100 μM non-template DNA (ntDNA) in reconstitution buffer RB (10 mM Tris HCl pH 8.0, 40 mM KCl, 5 mM MgCl_2_). Afterwards, the annealing was done in a PCR thermocycler (95 °C 2 min, 75 °C 2 min, 45 °C 5 min, 45 °C and −2 °C/2 min for 20 cycles, 4 °C). The complex was formed by mixing nucleic acid scaffold (12 μM), RNAP (4 μM), DTT (1 mM), BSA (0.02 mg/ml) and with or without full-length NusG (80 μM), followed by 30 min incubation at 37 °C. The samples were separated on the 4-15% native polyacrylamide gel (400 V for 10 min at 10 °C, then 45 min with 2.5 W constant power at 10 °C) and visualised by staining with Ethidium bromide and Coomassie blue.

### RNA extension assay

To confirm that the nucleic acid scaffold, which was used in EM reconstructions, does not induce pausing, the RNA was first 5’-labelled with ^32^P-γ-ATP using T4 polynucleotide kinase (NEB) according to the manufacturer’s protocol. The nucleic acid scaffold was reconstituted using a 2-fold molar excess of ntDNA and tDNA over RNA (5 μM final RNA concentration). tDNA and RNA were first mixed in reconstitution buffer (RB, 10 mM Tris-HCl, pH 8.0, 40 mM KCl, 5 mM MgCl_2_), incubated for 2 min at 98 °C, and then slowly cooled to room temperature in a water bath. The elongation complex was formed by mixing the RNAP (1000 nM) with nucleic acid scaffold (500 nM) and combinations of different transcription factors (NusA and/or NusG, 4 μM final concentration each, see Supplementary Fig. 1a) in EM buffer supplemented with 0.02 mg/mL acetylated BSA, and incubated at 37 °C for 2 min. ntDNA was added (1000 nM) and the sample was incubated for another 13 min at 37 °C. One base RNA extension assays were started at room temperature by adding UTP (10 μM) and samples were taken at regular time intervals (7”, 14”, 21”, 30”, 1’, 5′) and mixed with stop buffer (8 M urea, 20 mM EDTA pH 8.0, 5 mM Tris-HCl pH 7.5, 0.5% bromophenol blue and 0.5% xylene cyanol). UTP (1 mM) was added to the final sample to drive the reaction to completion for 10 min (chase). The RNA products were separated by denaturing urea PAGE (15% polyacrylamide and 7 M urea; 1× TBE) run at 50 W for 3 hours. Following electrophoresis, the gels were dried on a gel dryer under vacuum at 70 °C for 1 hour. The gel was exposed overnight to a storage phosphor screen and visualised using a PhosphorImager (Typhoon 8600). The results were quantified by ImageQuant (GE Healthcare, version 5.2). Each RNA product was quantified as a fraction of total RNA per lane and corrected for inactive RNAP (RNA remaining in the chase lane). The extension reaction curve was fitted by nonlinear regression of RNA versus time (http://plasma-gate.weizmann.ac.il/Grace/) using a double exponential decay.

### ops-pause assay

For the *ops*-*pause* assay, the template DNA contained a T7A1 promoter followed by a U-less region (A48) and the pause site (*ops*). Linear template DNA was generated by PCR amplification followed by agarose gel purification. RNAP holoenzyme was assembled by mixing RNAP (60 nM) with sigma70 (300 nM) and 0.02 mg/ml acetylated BSA in EM buffer (10 mM HEPES pH 8.0, 150 mM KOAc, 2 mM DTT, 10 μM ZnCl_2_, 5 mM Mg(OAc)_2_), and incubated at 37 °C for 10 min. Initiation complexes were formed by incubating RNAP core enzyme, sigma70 and template DNA (50 nM) at 37 °C for 10 min. Elongation complex (EC) halted at A48 was formed by mixing ApU primer (100 μM), ATP (20 μM), GTP (20 μM) and ^32^P-α-CTP (0.3 μM) at 37 °C for 2 min. After the incubation, NusG (200 nM), NusA (300 nM) or elongation buffer was added, and the transcription resumed at 4 °C with addition of all four rNTPs (150 μM) and competitor DNA encoding a full-consensus promoter sequence to ensure single round transcription (*fullcon*, 1 μM). Samples were taken at regular time intervals (10”, 20”, 30”, 1’, 1’ 30”, 2’, 3’, 5′, 10’) and mixed with stop buffer (8 M urea, 20 mM EDTA pH 8.0, 5 mM Tris-HCl pH 7.5, 0.5% bromophenol blue and 0.5% xylene cyanol). For chase, rNTPs (5 mM) were added to the remaining reactions and incubated for an additional 5 min. RNA products were separated (10% polyacrylamide and 7 M urea; 1× TBE), visualised and quantified as described above. Each RNA species at the ops-site was quantified as a fraction of total RNA per lane. The rate of pause escape was determined by nonlinear regression of the paused RNA species versus time (http://plasma-gate.weizmann.ac.il/Grace/) using a double exponential decay.

### his-pause assay

To monitor the effect of NusG with or without NusA on the *his-pause* elongation complex (his-PEC) a previously published experimental setup was used^7^. The same nucleic acid scaffold was prepared by the annealing procedure described before^7^. The elongation complex was formed by mixing the RNAP (1000 nM) with nucleic acid scaffold (500 nM) in EM buffer supplemented with 0.02 mg/mL acetylated BSA at 37 °C for 10 min. The 27nt-RNA in the complex was first radioactively labelled by incorporation of ^32^P-α-CTP (30 mCi) at 37 °C for 1 minute, followed by addition of non-radioactive CTP (2 μM final) and UTP (100 μM final), extending the RNA to 29nt length. The his-PEC was then incubated with TFs at 37 °C for 2 min, in the following mixtures: 1) RNAP; 2) RNAP + NusA; 3) RNAP + NusG; 4) RNAP + NusA + NusG; 5) RNAP + NusA + NGN. To follow the pause-escape over time, once the next nucleotide GTP (10 μM) was added at room temperature, samples were taken at regular time intervals (7″, 14″, 21″, 30″, 45″, 1′, 1′30″, 2′, 2′30″, 3′, 5′, 10′, 15′) and mixed with stop buffer. At the end GTP (1 mM) was added to drive the reaction to completion for 5 min (chase). RNA products were separated (15% polyacrylamide and 7 M urea; 1× TBE), visualised and quantified as described above. Each RNA species was quantified as a fraction of total RNA per lane and corrected for inactive RNA remaining in the chase lane. The rate of pause escape was determined by nonlinear regression of the paused RNA species versus time (http://plasma-gate.weizmann.ac.il/Grace/) using a double exponential decay.

### Rho-termination assay

The analysis of the termination efficiency on the tR1 terminator were performed on a template DNA, based on previous studies^31,37–40^. A part of the *cro* gene and the *rho*-terminator sites from the lambda bacteriophage genome were cloned into pIA171 vector and verified by sequencing. The template DNA containing T7A1 promoter, U-less region (A29), *cro* gene, rutA, boxB, rutB and termination regions were amplified by PCR and agarose gel-purified. The termination assays were performed as follows: holoenzyme was prepared by mixing RNAP (29 nM) with sigma70 (146 nM) and 0.02 mg/ml acetylated BSA in EM buffer at 37 °C for 10 min. The open complex was formed by mixing the holoenzyme with template DNA (35 nM) at 37 °C for 10 min. Halted complex at A29 (a 29nt long U-less RNA) was formed by mixing ApU primer (17.5 μM), ATP (14 μM), GTP (14 μM) and ^32^P-α-CTP (84 nM) at 37 °C for 2 min. Six mixtures of the complexes were made to assess the effect of NusA and NusG: 1) RNAP; 2) RNAP + Rho; 3) RNAP + NusG + Rho; 4) RNAP + NusA + Rho; 5) RNAP + NusA + NusG + Rho; 6) RNAP + NusA + NGN + Rho. The complexes were formed by incubating 44 nM Rho with NusG/NGN (88 nM), NusA (146 nM), or EM buffer for a few minutes. Transcription was restarted at room temperature in the presence of rNTPs (760 μM) and *fullcon* promoter DNA (1 μM). Samples were taken after indicated time intervals (10”, 20”, 30”, 1’, 2’, 5′, 10’, chaise) and the reactions were stopped by mixing with equal volume of stop buffer. At the end, rNTPs (5 mM) were added to drive all reactions to completion for 5 min. RNA products were separated and visualised as described above. Termination efficiencies were calculated as the ratios of the total terminated RNA products (or the terminated RNA products at different termination site) over the sum of RNA that has reached the termination site or bypassed it.

### Mathematical modelling applied to the Rho-termination assay

To follow the termination reaction, we quantified the RNA product of the early termination region at each interval time (10″, 20″, 30″, 1′, 2′, 5′, 10′, chase). The RNA species were normalised using a low-molecular-weight reference band in each lane that was constant over time and not influenced by the termination. Experiments were done in triplicates to estimate errors. At *t* = 0, transcription initiates but the population of RNAPs become asynchronous over time before reaching the terminator. The flux of RNAP reaching the termination site at *t* = *t*_*0*_ was modelled by a Gaussian peak of width *σ*. Therefore, the flux d*R*/d*t* at the termination site of a unit amount of RNAP can be modelled by

1 $${{{{{{\mathrm{d}}}}}}R}/{{{{{{\mathrm{d}}}}}}t}\,=\,\frac{1}{\sqrt{2\,\pi \,\sigma }}{{\exp}}\left[-\frac{{(t-{t}_{0})}^{2}}{2\,{\sigma }^{2}}\right]$$

To take into account that RNAP can resume transcription after pausing, Eq. (1) is modified as follows

2 $${{{{{{\mathrm{d}}}}}}R}/{{{{{{\mathrm{d}}}}}}t}\,=\,\frac{1}{\sqrt{2\,\pi \,\sigma }}{{\exp }}\left[-\frac{{(t-{t}_{0})}^{2}}{2\,{\sigma }^{2}}\right]-\frac{R(t)}{{\tau }_{{{{{{{\mathrm{pause}}}}}}}}}$$

where$\,{\tau }_{{{{{{{\mathrm{pause}}}}}}}}$ is the average pausing time. Since not all RNAPs stop transcription at the early termination site we introduced a probability *P*_off_. If all RNAPs terminate (*P*_off_ = 1), the band intensities accumulate and reach a plateau at the termination site, derived from Eq. (1) (Supplementary Fig. 6c, plot I). On the contrary, if all RNAPs bypass the termination site (*P*_off_ = 0), the band intensities at the termination site are derived from Eq. (2) (Supplementary Fig. 6c, plot II) and this would manifest itself as a peak of intermediate RNA products, which then gradually decreases to basal level. In reality, the reaction proceeds as an intermediate of the two extremes: a fraction of RNAPs terminate with an intermediate probability (0 < *P*_off_ < 1) and the remaining paused fraction (1$-$*P*_off_) may eventually continue transcription after an average time$\,{\tau }_{{{{{{{\mathrm{pause}}}}}}}}$ (illustrated in Supplementary Fig. 6c, plots III and IV). Finally, the function used to fit the experimental data is

3 $$R(t)\,=A\,[P_{{{{{{\rm{off}}}}}}}R_{1}(t)\,+\,(1-P_{{{{{{\rm{off}}}}}}})R_{2}(t)]+R_{0}$$

with

$${R}_{1}(t)\,=\,\left({{{{{\rm{Erf}}}}}}\frac{t-{t}_{0}}{\sqrt{2}\,\sigma }\,+{{{{{\rm{Erf}}}}}}\frac{{t}_{0}}{\sqrt{2}\,\sigma }\right)/2\,\,{{{{{\rm{and}}}}}}$$

$${R}_{2}(t)\,=\,-{{\exp }}\left(\frac{{\sigma }^{2}-2(t-{t}_{0})\,{\tau }_{{{{{{{\mathrm{pause}}}}}}}}}{2{\tau }_{{{{{{{\mathrm{pause}}}}}}}}^{2}}\right)\left({{{{{\rm{Erf}}}}}}\frac{{\sigma }^{2}-(t-{t}_{0}){\tau }_{{{{{{{\mathrm{pause}}}}}}}}}{\sqrt{2}\,\sigma {\tau }_{{{{{{{\mathrm{pause}}}}}}}}}\,-{{{{{\rm{Erf}}}}}}\frac{{\sigma }^{2}+{t}_{0}{\tau }_{{{{{{{\mathrm{pause}}}}}}}}}{\sqrt{2}\,\sigma {\tau }_{{{{{{{\mathrm{pause}}}}}}}}}\right)/2$$

being respectively the solutions of differential Eqs. (1) and (2) (obtained with *Mathematica 11.1* from Wolfram Research) with Erf = Error function. In Eq. (3) $A$ is a coefficient, which reflects the amount of paused RNAP and ${R}_{0}$ is the value of ${R}(t)$ at *t* = 0 (background signal). Only the variations of $A$ relative to the value for RNAP alone are meaningful (Supplementary Fig. 6d). The fitting was done in two steps to make the procedure stable and avoid influencing the result by the strong correlation between $A$ and ${P}_{{{{{{\rm{off}}}}}}}$ . First, the correlation coefficient between the experimental and theoretical data calculated with $A$ = 1 and ${R}_{0}$ = 0 was maximised (since this correlation coefficient is independent of $A$ and ${R}_{0}$). Second, $A$ and ${R}_{0}$ were determined by a simple linear least-squares method with all other free parameters having their values from correlation maximisation. Parameter errors were estimated from their variations observed from repeating the fitting procedure many times with experimental data being randomly modified within the limits of the error bars.

### Ladder for Rho-termination assay

To better identify the termination sites, we prepared a radioactively labelled DNA marker to estimate the size of the RNA species. pBR322 vector (1 mg/ml) was digested with restriction enzyme MspI (NEB, #N3032S). 5′-phosphates were removed by adding 20 U of calf intestinal phosphatase (NEB) and the vector fragments were purified by standard phenol/chloroform extraction and alcohol precipitation. In the end the fragments were labelled with ^32^P-γ-ATP using T4 polynucleotide kinase (NEB) and stored at −20 °C.

### Sample preparation

To prepare complexes for cryo-EM study, the ntDNA, tDNA and RNA oligonucletides were mixed in 2:2:1 molar ratio and annealed in a PCR thermocycler (2 min at 95 °C, 2 min at 75 °C, 5 min at 45 °C, −2 °C/min over 20 min, keep at 4 °C) in reconstitution buffer (10 mM Tris-HCl pH 8.0, 40 mM KCl, 5 mM MgCl_2_). The RNAP-NusA-NusG complex was formed by mixing RNAP, nucleic acid scaffold, NusG and NusA in 1:2:3:3 molar ratio in EM buffer (10 mM HEPES pH 8.0, 150 mM KOAc, 2 mM DTT, 10 μM ZnCl_2_, 5 mM Mg(OAc)_2_), incubated at 37 °C for 5–10 min. For the other two complexes, RNAP-NusA and RNAP-NusG, the components were mixed with the same molar ratios. Each complex was purified by gel filtration (Superose 6 10/300 GL). The complex was then concentrated to 7-10 mg/ml using Amicon Ultra centrifugal filter units. CHAPSO (3-([3-Cholamidopropyl]dimethylammonio)−2-hydroxy-1-propanesulfonat) was added to the sample at 8 mM final concentration just before grid freezing. C-flat grids (CF-1.2/1.3 400 mesh holey carbon) were glow-discharged with ELMO^TM^ glow discharge system (Cordouan Technologies) for 30 s at 2.5 mA. 4 μl of samples were applied on the grids and plunge-frozen in liquid ethane using a Vitrobot Mark IV (FEI) with 95% chamber humidity at 10 °C (blot force 6, blotting time 2 s).

### Cryo-EM data collection and processing

Images were recorded on different Titan Krios (FEI) microscopes controlled by SerialEM (ver. 3.6) operated at 300 keV acceleration voltage and each equipped with a K2 Summit camera (Gatan, Inc., Pleasanton, CA) placed at the end of a GIF Quantum energy filter (Gatan, Inc.) in zero-energy-loss mode with a slit width of 20 eV. The target defocus ranges were set to −0.8 to −3 μm. The movies contained 40 frames, which were collected in super-resolution counting mode with super-resolution pixel size of 0.55 or 0.52 Å/pixel, and exposures ranging from 5 to 6.4 e^−^/A^2^/s, which corresponds to a total dose of ~50 e^−^/A^2^.

The images were first motion-corrected and dose weighted using Motioncor2^41^. The pixel size of micrographs was rescaled with relion_image_handler to 1.09 Å/pixel in order to combine datasets collected on different microscopes. The contrast transfer function (CTF) for each micrograph was then estimated by CTFFIND4^42^ or Gctf^43^. The particle-picking templates were generated using semi-automated swarm method in EMAN2^44^. Automatic particle picking was done on the lowpass-filtered (20 Å) and contrast-inverted micrographs by using Relion^45,46^. The particles were extracted from the dose-weighted micrographs and the resulting datasets from the same complex were merged. Joined particles were re-extracted with 4×4 binning and further sorted by 2D-classification. Selected particles were re-extracted with 1 × 1 binning and used for 3D-refinement using an ab-initio model generated in cryoSPARC^22^ and 3D-classification in Relion. Further 3D-refinement, heterogenous refinements and 3D-variability analysis were performed in cryoSPARC. 3D-variability analysis as implemented in cryoSPARC allows modelling of the conformational landscape of a molecule based on the experimental cryo-EM data^47^.

### Structural modelling

We constructed an initial model of the different complexes by combining the cryo-EM structure of *E. coli* RNAP (PDB ID: 6ALH)^48^, a previously published structure of a paused *E. coli* RNAP (PDB ID: 6FLQ)^7^ and the NMR structure of *E. coli* NusG (PDB ID: 2K06)^8^. UCSF Chimera^49^ was used for initial model placement into the EM maps. Model building included real-space refinement using the Phenix^50^ software suite and manual modifications in Coot^51^. Furthermore, the upstream and downstream DNA duplex were built de novo in Coot. The resulting models were real-space refined using secondary structure restraints and geometry optimisation in Phenix against density maps sharpened by applying DeepEMhancer^52^ with default settings. The interaction of NusG with RNAP was estimated in ChimeraX (Supplementary Fig. 2b, right panel).

### Sequences of DNA templates used in transcription assays measuring the *ops* pause

A linear template DNA was generated by PCR from the pIA171_A48_*ops-site* plasmid and gel-purified for the *ops* pause assay with the T7A1 promotor (contains −35 and −10 elements), the A48 region and the *ops* pause sequences (in capital) (Supplementary Fig. 7a):

5′ttgacttaaagtctaacctataggatacttacagccatcgagagggacacggggaaacaccagggacacggggaaacaccaccatGGCGGTAGCGTGCgagatctccgtctatcaaactcaacgaccccttccttctccccatcgctacctcatatccgcacctcctcaaacgctacctcgaccagcctccctccctccctctagacctgcaggcatgcgagagtagggaactgccaggcatcaaagaaaacgaaaggcacagtcgaaagac3′

A linear template DNA was generated from pIA171_A29_*cro*_rutA_boxB_rutB_*rho*_terminator plasmid for the termination assay, with the T7A1 promotor (contains −35 and −10 elements), the A29 region, the rutA_boxB_rutB and the *rho*_terminator (capital) sequences (Supplementary Fig. 7b):

5′-ttgacttaaagtctaacctataggatacttacagccatcgagagggacacggggaaacaccaccatggatctcggcgtatatcaaagcgcgatcaacaaggccattcatgcaggccgaaagatttttttaactataaacgctgatggaagcgtttatgcggaagaggtaaagcccttcccgagtaacaaaaaaacaacagcataaataaccc*cgctcttacacattccagccctgaaaaagggcatcaaattaaaccacacctatg*gtgtatgCATTTATTTGCATACATTCAATCAATTGTTATCTAAGGAAATACTTACATATGGTTCGTGCaaacaaacgcaacgaggcttctagacctgcaggcatgcgagagtagggaactgccaggcatcaaagaaaacgaaaggcacagtcgaaagac-3′

The DNA and RNA oligonucleotides for the *his* pause assay were:

tDNA_his-pause: 5′﻿-ctctgaatctcttccagcacacatcgggacgtactgacc-3′;

ntDNA_his-pause: 5′-ggtcagtacgtcccgtcgatcttcggaagagattcagag-3′;

27nt-RNA_his-pause: 5′-ccugacuagucuuucaggcgaugugug-3′

The DNA and RNA oligonucleotides for the EM studies (for all three complexes) were:

tDNA: 5′-ctctgaatctcttccgacgcgccgcgggacgtactgacc-3′;

ntDNA: 5′-ggtcagtacgtcctatcgatcttcggaagagattcagag-3′;

RNA: 5′-gaguccgcggcgcg-3′

### Reporting summary

Further information on research design is available in the Nature Research Reporting Summary linked to this article.

## Supplementary information

Supplementary information

Description of additional Supplementary File

Supplementary Movie 1

Supplementary Movie 2

Reporting Summary

## Data Availability

The data that support this study are available from the corresponding author upon reasonable request. The accession numbers for the eleven cryo-EM reconstructions (RNAP-EC non-swivelled, RNAP-EC swivelled, NusG-EC consensus, NusG-EC non-swivelled, NusG-EC swivelled, NusA-EC consensus, NusA-EC non-swivelled, NusA-EC swivelled, NusA-NusG-EC consensus, NusA-NusG-EC non-swivelled and NusA-NusG-EC swivelled) reported in this paper are EMD-13746, EMD-13745, EMD-13707, EMD-13716, EMD-13706, EMD-13709, EMD-13717, EMD-13718, EMD-13713, EMD-13714 and EMD-13715, respectively. Fitted models were deposited ﻿in the RCSB Protein Data Bank (https://www.rcsb.org/) with accession codes 7Q0K (RNAP-EC non-swivelled), 7Q0J (RNAP-EC swivelled), 7PY1 (NusG-EC consensus), 7PY8 (NusG-EC non-swivelled), 7PY0 (NusG-EC swivelled), 7PY3 (NusA-EC consensus), 7PYJ (NusA-EC non-swivelled), 7PYK (NusA-EC swivelled), 7PY5 (NusA-NusG-EC consensus), 7PY6 (NusA-NusG-EC non-swivelled) and 7PY7 (NusA-NusG-EC swivelled). Source data are provided with this paper.

## References

[1] Landick R (2006). The regulatory roles and mechanism of transcriptional pausing. Biochem. Soc. Trans..

[2] Kwak H, Lis JT (2013). Control of transcriptional elongation. Annu. Rev. Genet..

[3] Larson MH (2014). A pause sequence enriched at translation start sites drives transcription dynamics in vivo. Science.

[4] Vvedenskaya IO (2014). Interactions between RNA polymerase and the ‘core recognition element’ counteract pausing. Science.

[5] Roberts JW, Shankar S, Filter JJ (2008). RNA polymerase elongation factors. Annu. Rev. Microbiol..

[6] Artsimovitch I, Landick R (2000). Pausing by bacterial RNA polymerase is mediated by mechanistically distinct classes of signals. Proc. Natl Acad. Sci. USA.

[7] Guo X (2018). Structural basis for NusA stabilized transcriptional pausing. Mol. Cell.

[8] Mooney RA, Schweimer K, Rösch P, Gottesman M, Landick R (2009). Two structurally independent domains of E. coli NusG create regulatory plasticity via distinct interactions with RNA polymerase and regulators. J. Mol. Biol..

[9] Turtola, M. & Belogurov, G. A. NusG inhibits RNA polymerase backtracking by stabilizing the minimal transcription bubble. *Elife* **5**, e18096 (2016). 10.7554/eLife.18096 PMC5100998 27697152

[10] Kang JY (2018). Structural basis for transcript elongation control by NusG family universal regulators. Cell.

[11] Said N (2021). Steps toward translocation-independent RNA polymerase inactivation by terminator ATPase p. Science.

[12] Hao Z (2021). Pre-termination transcription complex: structure and function. Mol. Cell.

[13] Cardinale CJ (2008). Termination factor Rho and its cofactors NusA and NusG silence foreign DNA in E. coli. Science.

[14] Burns CM, Richardson LV, Richardson JP (1998). Combinatorial effects of NusA and NusG on transcription elongation and Rho-dependent termination in Escherichia coli. J. Mol. Biol..

[15] Burns CM, Richardson JP (1995). NusG is required to overcome a kinetic limitation to Rho function at an intragenic terminator. Proc. Natl Acad. Sci. USA.

[16] Lawson MR (2018). Mechanism for the regulated control of bacterial transcription termination by a universal adaptor protein. Mol. Cell.

[17] Peters JM (2012). Rho and NusG suppress pervasive antisense transcription in Escherichia coli. Genes Dev..

[18] Schmidt A (2016). The quantitative and condition-dependent Escherichia coli proteome. Nat. Biotechnol..

[19] Mooney RA (2009). Regulator trafficking on bacterial transcription units in vivo. Mol. Cell.

[20] Krupp F (2019). Structural basis for the action of an all-purpose transcription anti-termination factor. Mol. Cell.

[21] Kang JY (2018). RNA polymerase accommodates a pause RNA hairpin by global conformational rearrangements that prolong pausing. Mol. Cell.

[22] Punjani A, Rubinstein JL, Fleet DJ, Brubaker MA (2017). CryoSPARC: algorithms for rapid unsupervised cryo-EM structure determination. Nat. Methods.

[23] Abdelkareem M (2019). Structural basis of transcription: rna polymerase backtracking and its reactivation. Mol. Cell.

[24] Zivanov, J. et al. New tools for automated high-resolution cryo-EM structure determination in RELION-3. *Elife* **7**, e42166 (2018). 10.7554/eLife.42166 PMC6250425 30412051

[25] Sevostyanova A, Belogurov GA, Mooney RA, Landick R, Artsimovitch I (2011). The β subunit gate loop is required for RNA polymerase modification by RfaH and NusG. Mol. Cell.

[26] Beuth B, Pennell S, Arnvig KB, Martin SR, Taylor IA (2005). Structure of a mycobacterium tuberculosis NusA-RNA complex. EMBO J..

[27] Said N (2017). Structural basis for λN-dependent processive transcription antitermination. Nat. Microbiol..

[28] Chan CL, Landick R (1993). Dissection of the his leader pause site by base substitution reveals a multipartite signal that includes a pause RNA hairpin. J. Mol. Biol..

[29] Toulokhonov I, Artsimovitch I, Landick R (2001). Allosteric control of RNA polymerase by a site that contacts nascent RNA hairpins. Science.

[30] Kyzer S, Kook SH, Landick R, Palangat M (2007). Direct versus limited-step reconstitution reveals key features of an RNA hairpin-stabilized paused transcription complex. J. Biol. Chem..

[31] Lau LF, Roberts JW, Wu R (1982). Transcription terminates at λt(R1) in three clusters. Proc. Natl Acad. Sci. USA.

[32] Yin Z, Kaelber JT, Ebright RH (2019). Structural basis of Q-dependent antitermination. Proc. Natl Acad. Sci. USA.

[33] Wang C (2020). Structural basis of transcription-translation coupling. Science.

[34] Andersen KR, Leksa NC, Schwartz TU (2013). Optimized E. coli expression strain LOBSTR eliminates common contaminants from His-tag purification. Proteins Struct. Funct. Bioinform..

[35] Subbarayan PR, Deutscher MP (2001). Escherichia coli RNase M, is a multiply altered form of RNase I. RNA.

[36] Vassylyeva MN (2002). Purification, crystallization and initial crystallographic analysis of RNA polymerase holoenzyme from Thermus thermophilus. Acta Crystallogr. Sect. D Biol. Crystallogr..

[37] Chen CY, Richardson JP (1987). Sequence elements essential for rho-dependent transcription termination at lambda tR1. J. Biol. Chem..

[38] Graham JE, Richardson J (1998). P. rut Sites in the nascent transcript mediate rho-dependent transcription termination in vivo. J. Biol. Chem..

[39] Vieu E, Rahmouni AR (2004). Dual role of boxB RNA motif in the mechanisms of termination/ antitermination at the lambda tR1 terminator revealed in vivo. J. Mol. Biol..

[40] Graham JE (2004). Sequence-specific Rho-RNA interactions in transcription termination. Nucleic Acids Res..

[41] Zheng SQ (2017). MotionCor2: anisotropic correction of beam-induced motion for improved cryo-electron microscopy. Nat. Methods.

[42] Rohou A, Grigorieff N (2015). CTFFIND4: Fast and accurate defocus estimation from electron micrographs. J. Struct. Biol..

[43] Zhang K (2016). Gctf: Real-time CTF determination and correction. J. Struct. Biol..

[44] Carragher B (2007). EMAN2: An extensible image processing suite for electron microscopy. J. Struct. Biol..

[45] Scheres SHW (2012). RELION: implementation of a Bayesian approach to cryo-EM structure determination. J. Struct. Biol..

[46] Nakane T, Kimanius D, Lindahl E, Scheres SHW (2018). Characterisation of molecular motions in cryo-EM single-particle data by multi-body refinement in RELION. Elife.

[47] Punjani A, Fleet DJ (2021). 3D variability analysis: resolving continuous flexibility and discrete heterogeneity from single particle cryo-EM. J. Struct. Biol..

[48] Kang, J. Y. et al. Structural basis of transcription arrest by coliphage HK022 nun in an escherichia coli rna polymerase elongation complex. *Elife* **6**, e25478 (2017). 10.7554/eLife.25478 PMC5386594 28318486

[49] Pettersen Ef Fau-Goddard TD (2004). UCSF Chimera–a visualization system for exploratory research and analysis. J. Comput. Chem..

[50] Liebschner D (2019). Macromolecular structure determination using X-rays, neutrons and electrons: recent developments in Phenix. Acta Crystallogr. Sect. D Struct. Biol..

[51] Emsley P, Lohkamp B, Scott WG, Cowtan K (2010). Features and development of Coot. Acta Crystallogr. Sect. D Biol. Crystallogr..

[52] Sanchez-Garcia R (2021). DeepEMhancer: a deep learning solution for cryo-EM volume post-processing. Commun. Biol..

