# Supplementary material for: Transcription factors modulate RNA polymerase conformational equilibrium
Source: Nat Commun. 2022 Mar 22;13:1546. doi: 10.1038/s41467-022-29148-0 (PMC8940904; doi:10.1038/s41467-022-29148-0)
Supplement: Supplementary file 1 — Supplementary information [file 41467_2022_29148_MOESM1_ESM.pdf]

Supplementary Information

Transcription factors modulate RNA Polymerase conformational equilibrium

Chengjin Zhu[1,2,3,4], Xieyang Guo[1,2,3,4,5], Philippe Dumas[1,2,3,4], Maria Takacs[1,2,3,4], Mo'men Abdelkareem[1,2,3,4], Arnaud Vanden Broeck[1,2,3,4], Charlotte Saint-André[1,2,3,4], Gabor Papai[1,2,3,4], Corinne Crucifix[1,2,3,4], Julio Ortiz[1,2,3,4,6], Albert Weixlbaumer[1,2,3,4,*]

[1]Department of Integrated Structural Biology, Institut de Génétique et de Biologie Moléculaire et Cellulaire (IGBMC)

[2]Université de Strasbourg

[3]CNRS UMR7104

[4]INSERM U1258, 67404 Illkirch Cedex, France

[5]present address: GlaxoSmithKline, Gunnels Wood Road, Stevenage, Herts SG1 2NY, United Kingdom

[6]present address: Forschungszentrum Jülich, Ernst Ruska-Centre for Microscopy and Spectroscopy with Electrons, Jülich, Germany

[*]Correspondence to: albert.weixlbaumer@igbmc.fr

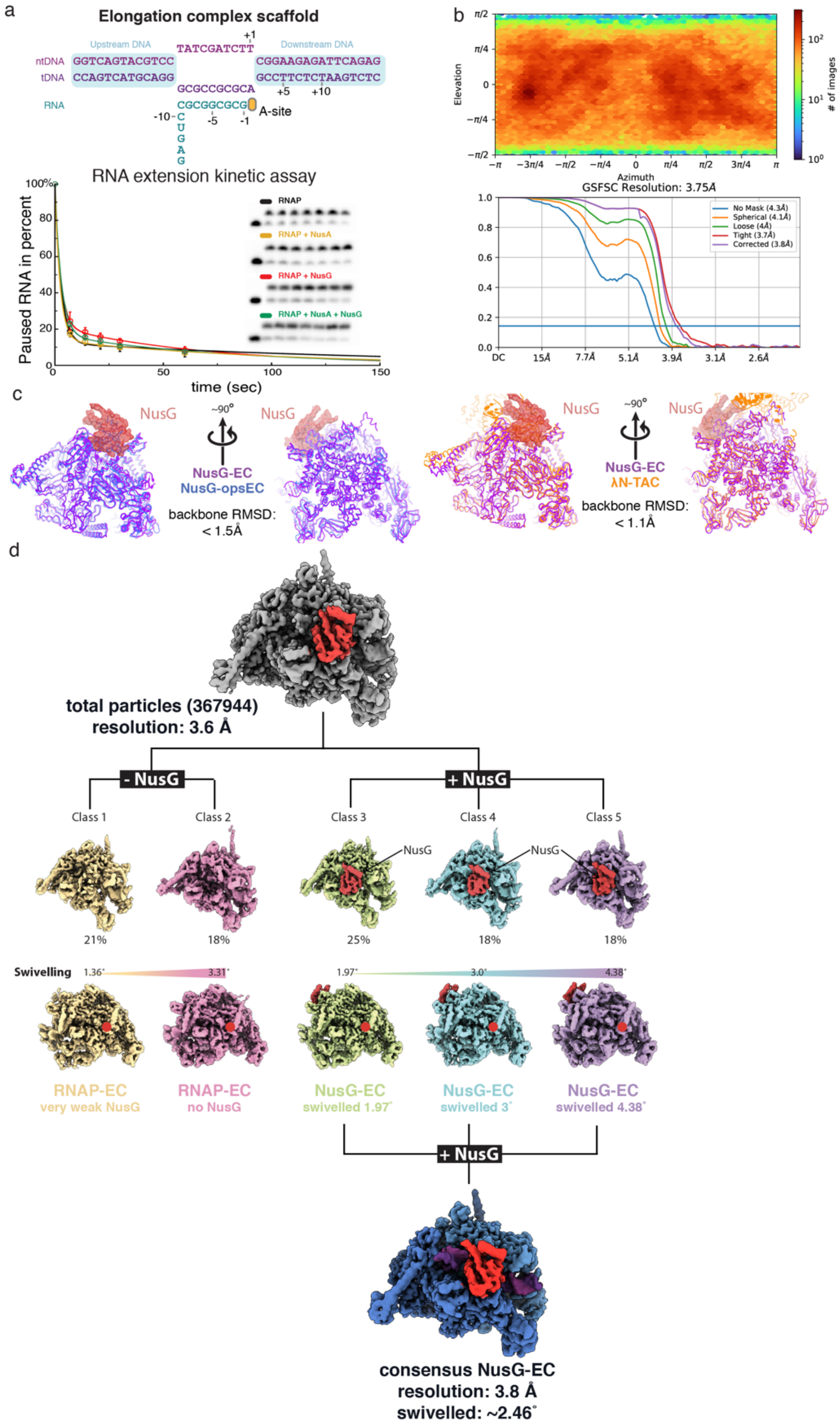

Supplementary Figure 1 (a) Schematics of the RNA-DNA scaffold (top) used for structural studies and RNA extension kinetics (bottom). Nucleic acid scaffold (top) used to form a canonical post-translocated state with 9 base pairs of the RNA-DNA hybrid and

an active site ready to bind a substrate (empty A site). The nucleic acid scaffold does not induce pausing as evidenced by fast RNA extension kinetics. Extension rates and half-lives cannot be measured reliably (bottom). RNA was quantified in triplicates (n=3) and error bars indicate standard deviations from the mean. The source data are provided as a Source Data file. **(b)** Distribution of particle orientation shows no preferential orientation bias. Fourier shell correlation (FSC) plot for half-maps with 0.143 FSC criteria indicated. The nominal resolution is 3.8 Å. **(c)** Comparison of NusG-EC (violet) and NusG-opsEC (blue, left) and NusG-EC (violet) and λN-TAC (orange, right) with aligned and superimposed structural models. Overall, the NusG-EC structure resembles the λN-TAC and NusG-opsEC complexes[10,20]. **(d)** Heterogenous refinements of NusG-EC in cryoSPARC. The refined structure with all particles (shown in grey on the top) has the highest nominal resolution (3.6 Å). The first two RNAP-EC classes (class 1 and 2) have very weak or no density for NusG. RNAP-EC class 2 lacks density for the upstream DNA. Three other classes (3 to 5) correspond to NusG-EC but with different extents of swivelling of RNAP. The rotation axis (red dot) and the rotation angles with respect to a substrate bound RNAP EC (PDB ID 6RH3) are indicated. Particles from these three classes were combined to obtain the consensus NusG-EC refinement in (b).

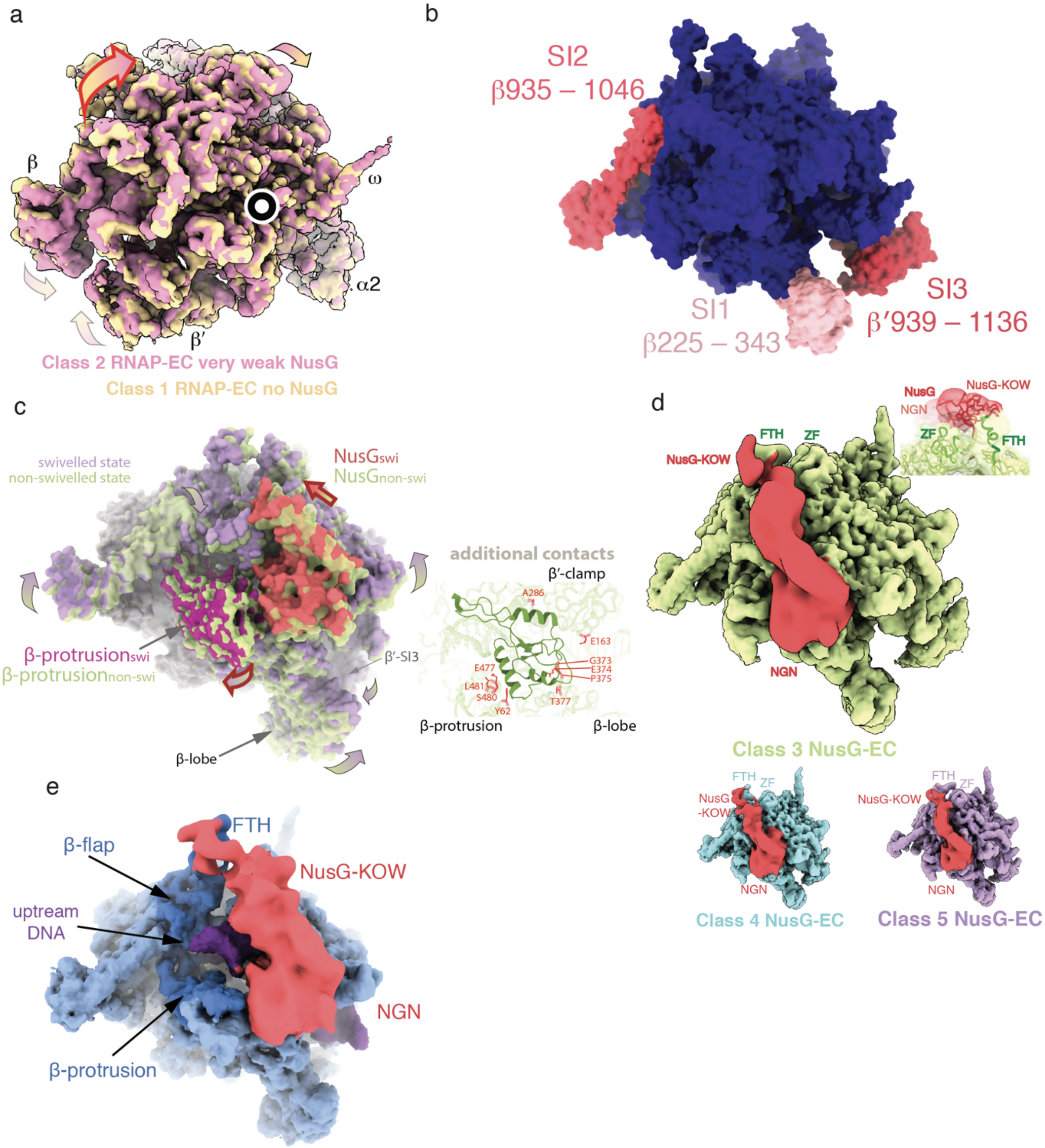

Supplementary Figure 2 (a) Conformational changes in classes lacking NusG. Class 1 and 2 were aligned based on the RNAP core module. The SI3 domain is closer to the β-lobe in the more swivelled conformation. The white and black circle designates the rotation axis. The red arrow indicates the swivel rotation. **(b)** The overall structure of RNAP is not rigid. Regions with the highest flexibility are highlighted and labelled: lineage specific insertions SI1, SI2 and SI3 show the largest differences compared to previous NusG containing structures[10,20]. **(c)** A structural superposition of non-swivelled and

swivelled conformations based on the RNAP core module (RNAP in non-swivelled conformation is in light green, RNAP in swivelled conformation is in purple and NusG in red). The β-protrusion (in dark purple) moves further away from NusG, while the β-lobe moves closer to the SI3 domain as a result of swivelling. In the inset, some potential additional contact points for NusG (in green) are shown when RNAP adopts non-swivelled state. β-protrusion (Y62, E477, S480, L481); β-lobe (G373, E374, P375, T377); β-clamp (E163, A286). **(d)** The flexible KOW domain of NusG. A map low-pass filtered to 10 Å indicated that the NusG-linker and NusG-KOW extend from the NGN and reach the FTH of RNAP. The NusG-KOW density appears on top of the FTH density. The results are similar for the two other NusG-EC classes (class 4 and class 5). **(e)** The upstream DNA is enclosed by NusG (red) and RNAP (regions of RNAP involved are depicted in dark blue). The map for NusG is low-pass filtered to 10 Å.

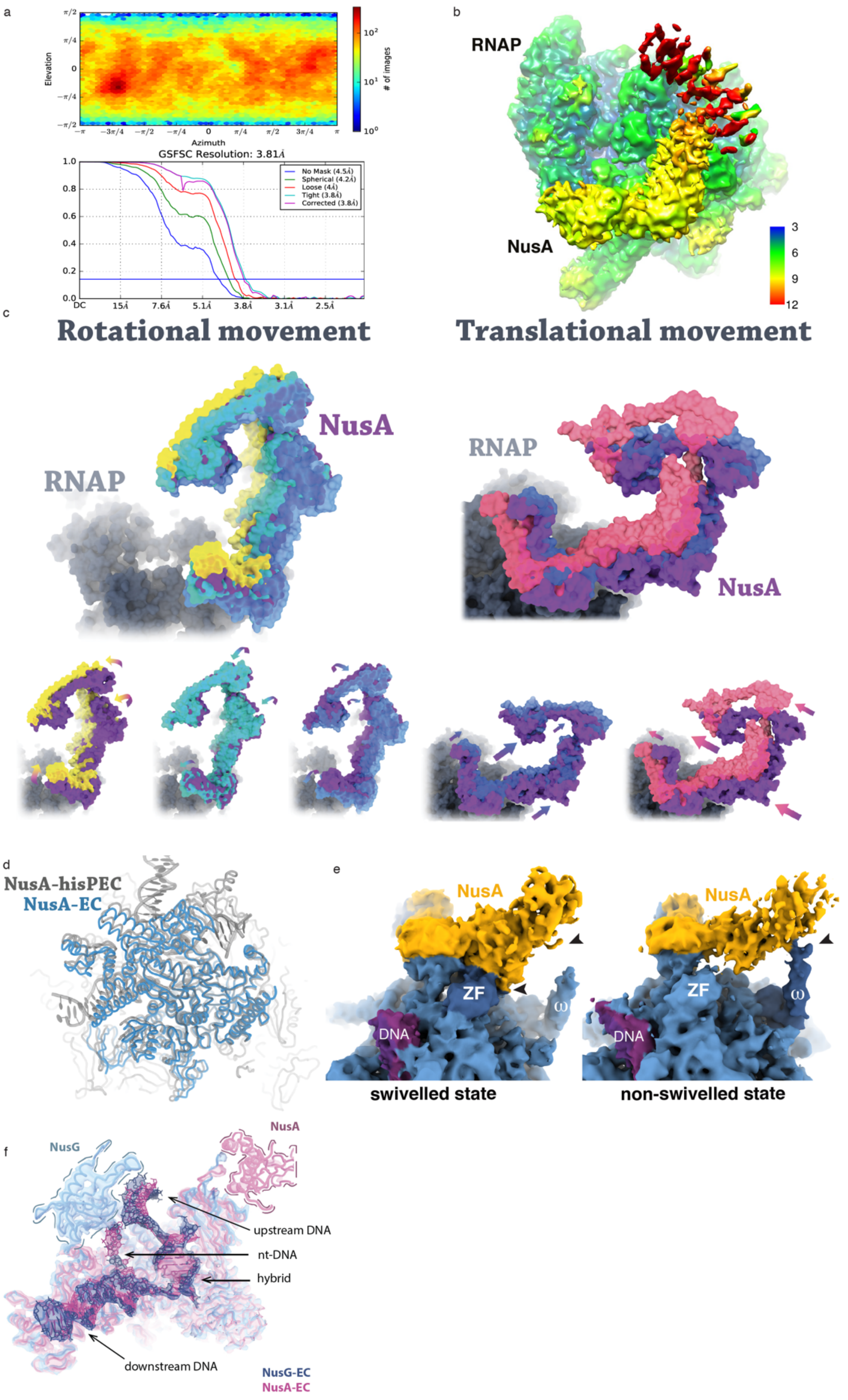

a
Elevation
Azimuth
# of images
GSFSC Resolution: 3.81Å
No Mask (4.5Å)
Spherical (4.2Å)
Loose (4Å)
Tight (3.8Å)
Corrected (3.8Å)
DC
15Å
7.6Å
5.1Å
3.8Å
3.1Å
2.5Å
b
RNAP
NusA
3
6
9
12
c
Rotational movement
Translational movement
NusA
RNAP
RNAP
NusA
d
NusA-hisPEC
NusA-EC
e
NusA
ZF
ω
DNA
swivelled state
NusA
ZF
ω
DNA
non-swivelled state
f
NusG
NusA
upstream DNA
nt-DNA
hybrid
downstream DNA
NusG-EC
NusA-EC

Supplementary Figure 3 (a) Distribution of particle orientation shows no preferential orientation bias. Fourier shell correlation (FSC) plot for half-maps with 0.143 FSC criteria indicated. The nominal resolution is 3.8 Å. **(b)** Local resolution estimation for the density of NusA. **(c)** Conformational heterogeneity of NusA. Structures obtained from 3D-variability analysis shown as surfaces represent NusA in different orientations relative to RNAP, which reflects its flexibility (Superimposed following alignment to RNAP). NusA can rotate or translate in several directions. NusA in violet is in the consensus conformation used as a reference for comparison. **(d)** Similarity between NusA-EC (blue) and NusA-his-PEC (grey). Overall, the NusA-EC is very similar to NusA-his-PEC. The swivel module of NusA-EC is less swivelled compared to NusA-his-PEC aligned based on the RNAP core module. **(e)** Density map showing that NusA is closer to the zinc finger (ZF) in the more swivelled conformation; NusA interacts with ω in the less swivelled conformation, and this interaction is broken in the swivelled state. **(f)** Comparison of nt-DNA and upstream DNA between NusA-EC and NusG-EC. Maps and atomic models of NusG-EC (blue) and NusA-EC (pink) are superimposed. The upstream DNA and nt-DNA are better defined in NusG-EC compared to NusA-EC.

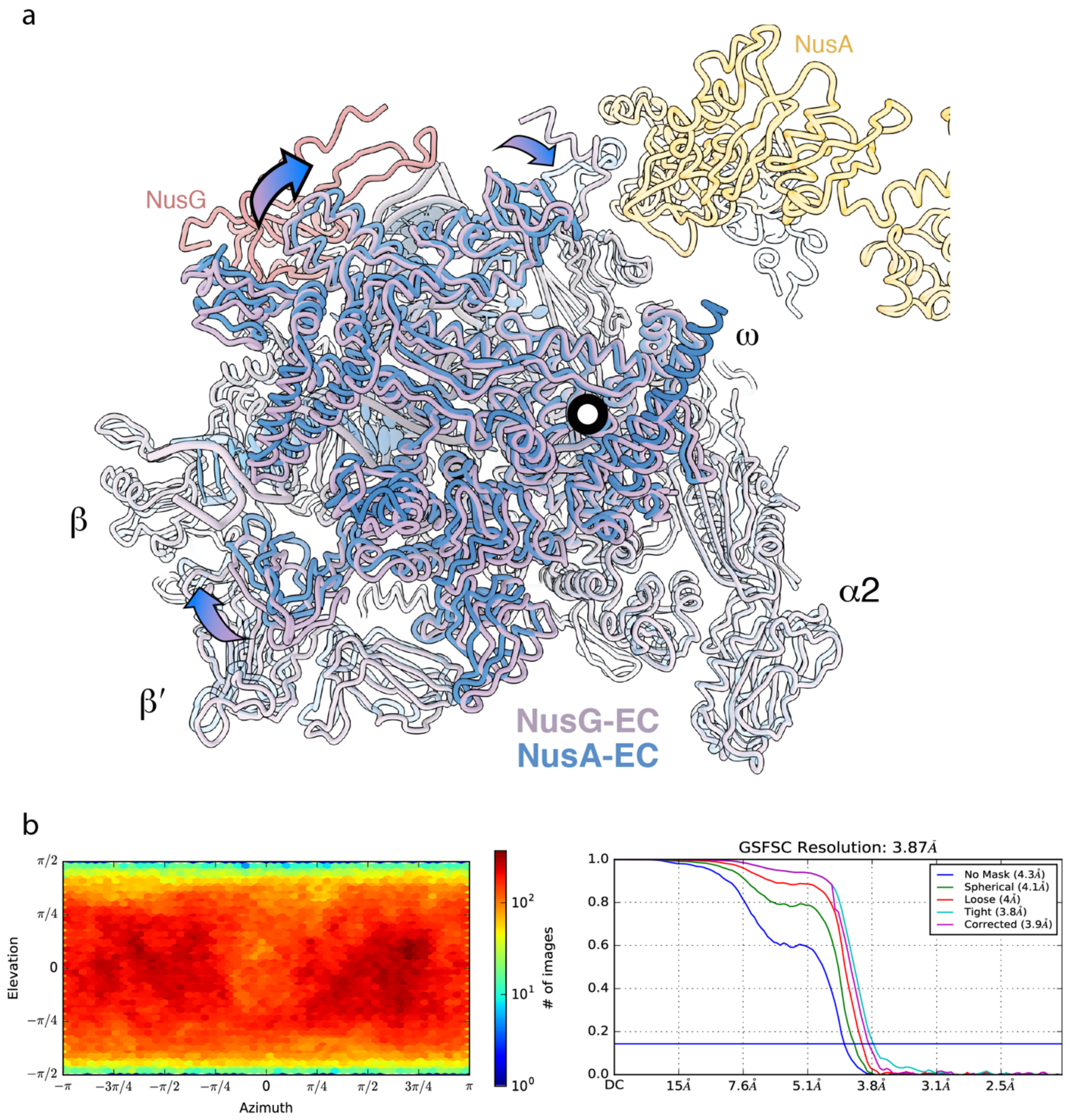

Supplementary Figure 4 (a) Comparison of most extreme swivelled conformation seen in 3D variability analysis for NusA-EC (6.5°) and NusG-EC (4.4°). The swivel module rotates more extensively in the NusA-EC. **(b)** Distribution of particle orientation of NusA-NusG-EC shows no preferential orientation bias. Fourier shell correlation (FSC) plot for half-maps with 0.143 FSC criteria indicated. The nominal resolution is 3.9 Å.

a

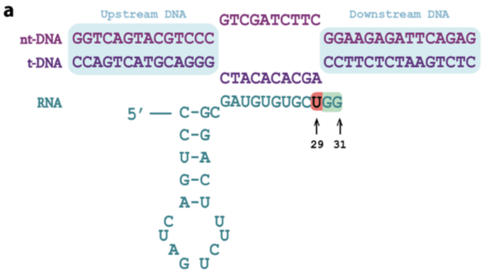

b

Equation used to fit experimental data

$$RNA(t) = RNA_{fast}*exp(-k_1*t)+(1-RNA_{fast})*exp(-k_2*t)$$

Reactions	RNAP	RNAP NusA	RNAP NusA NusG	RNAP NusA NGN	RNAP NusG
$RNAP_{fast}$ species	0.38	0.16	0.24	0.36	0.43
$RNAP_{slow}$ species	0.62	0.84	0.76	0.64	0.57
k_1(Pause-escape rate)	0.0149	0.0092	0.0186	0.0095	0.0194
k_2(Pause-escape rate)	0.0016	0.00069	0.00143	0.0011	0.0022
Half-life (fast species) (sec)	47 ±4.9	75 ±2.8	37 ±6.8	73 ±8	36 ±5
Half-life (slow species) (sec)	433 ±34	1005 ±0.5	485 ±34	632 ±58	315 ±36

c

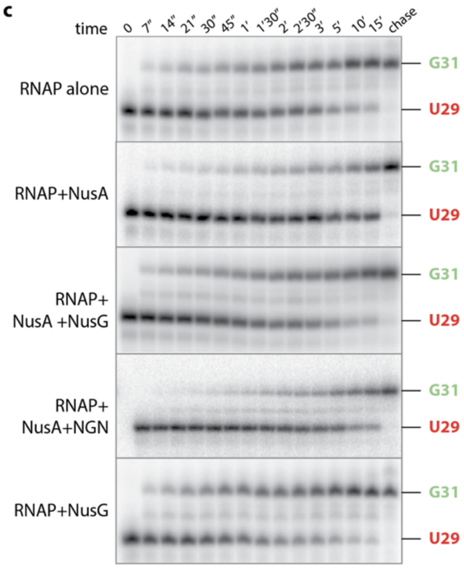

d

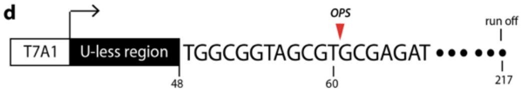

e

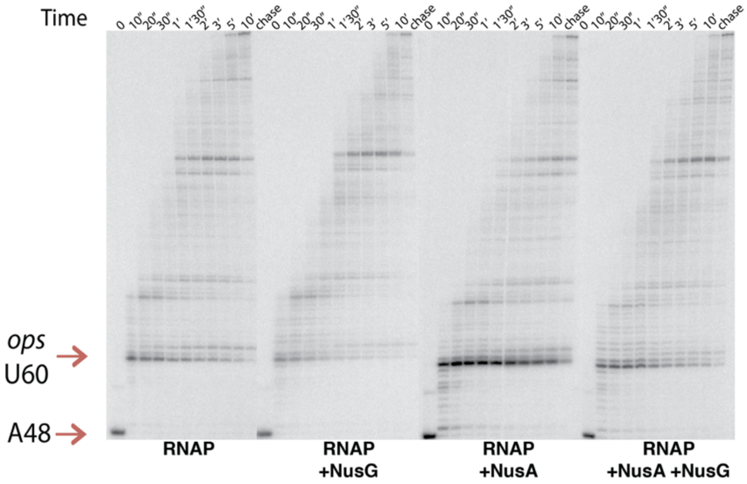

f

Equation used to fit experimental data

$$RNA(t) = RNA_{fast}*exp(-k_1*t)+RNA_{slow}*exp(-k_2*t)$$

Reactions	RNAP	RNAP NusG	RNAP NusA	RNAP NusA NusG
RNAPfast species at ops site	0.24	0.15	0.48	0.29
RNAPslow species at ops site	0.13	0.15	0.17	0.14
k_1 (Pausing escape rate)	0.13	0.09	0.08	0.08
k_2 (Pausing escape rate)	0.02	0.08	0.004	0.02
Half-life (fast species) (sec)	5 ±0.5	8 ±2	9 ±0.8	9 ±2
Half-life (slow species) (sec)	38 ±4.6	9 ±2	187 ±40	43 ±8.8

g Transcription factor effects at *ops*-pause

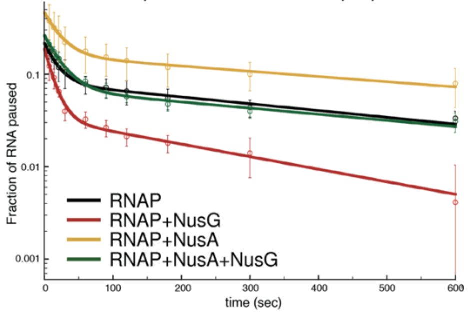

Supplementary Figure 5 (a) A schematic of the nucleic acid scaffold used in the *his* pause assay. A complex is extended to the U29 pause site, which is stabilized by the *his* RNA hairpin. Upon addition of GTP, RNAP escapes from the pause and incorporates two

nucleotides (RNA product at G31). **(b)** The kinetic parameters were obtained from fitting the experimental data using the equation shown above. **(c)** A representative 10% polyacrylamide urea gel shows the time course of pause escape +/- TF. **(d)** A schematic of the template DNA used in the *ops* pause assay. At time point 0, the complex was halted at A48 to synchronize the reaction. Elongation resumed upon addition of all rNTP substrates. RNAP paused at *ops*-pause site (U60). **(e)** A representative 10% polyacrylamide urea gel showing the time course for the ops-pause assay +/- TF. **(f)** The kinetic parameters were obtained from fitting the experimental data using the equation shown above. **(g)** Transcriptional pausing at the *ops*-pause was measured in single-round transcription assays (data reproduced from figure 4, y axis in log scale). For all panels, RNA was quantified in triplicates (n=3) and error bars indicate standard deviations from the mean. All the source data are provided as a Source Data file.

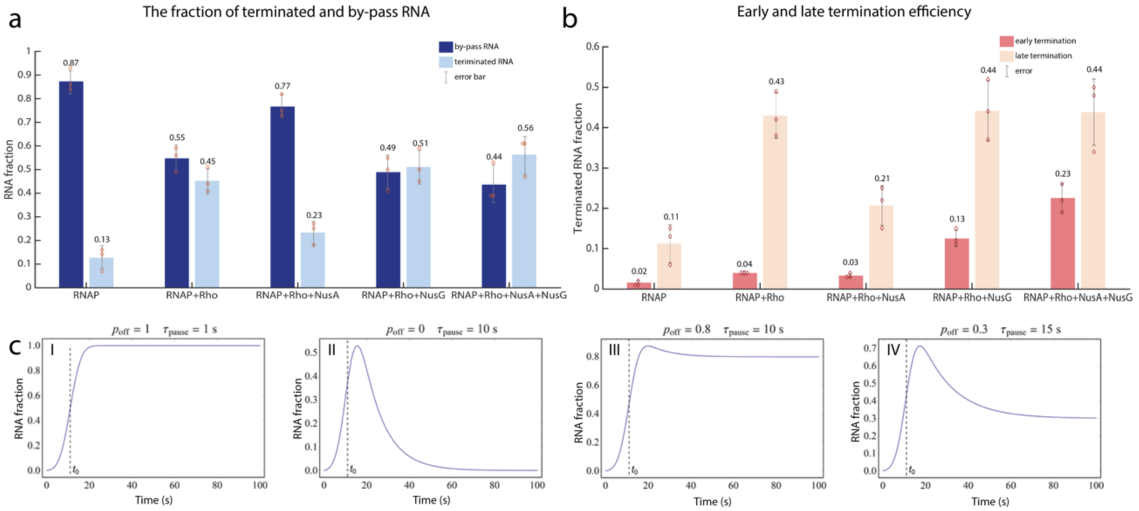

d

	A	P_{off} (%)	τ_{pause} (s)	σ (s)	t_0 (s)
**RNAP**	114 (37)	16.4 (2.9)	7.4 (1.3)	1.8 (1)	12 (1)
**RNAP+Rho**	103 (6)	22.2 (1.5)	7.9 (0.6)	0.84 (0.16)	12. (0.07)
**RNAP+Rho+NusA**	140 (2)	15.8 (0.4)	11.9 (0.3)	0.95 (0.04)	12. (0.02)
**RNAP+Rho+NusG**	63 (19)	77 (20)	*2.0* (*)	1.1 (0.6)	11.6 (1)
**RNAP+Rho+NusA+NusG**	117 (1)	100 (0.01)	*2.0* (*)	0.1 (0.46)	10.1 (0.6)

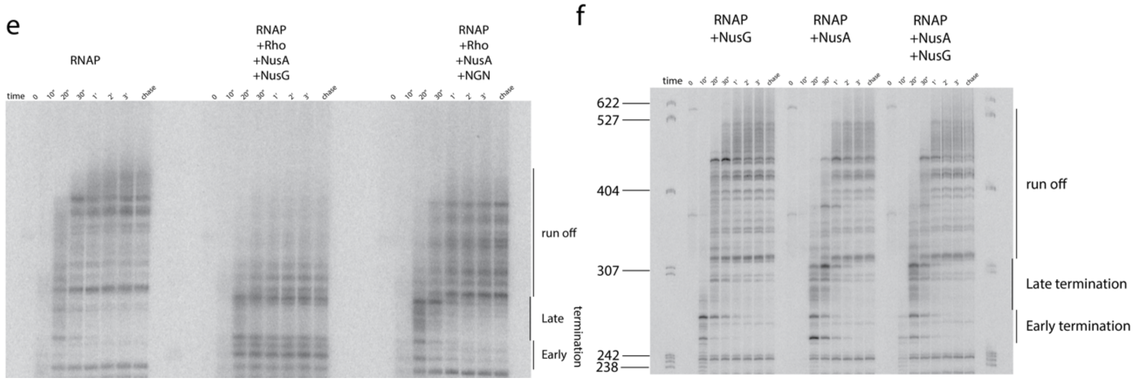

Supplementary Figure 6 (a) Quantification of RNA products at termination and run-off region. The cumulative termination efficiency (ratio of the terminated species over terminated and run-off) was determined for different combinations of TFs (indicated below the plot). RNA was quantified in triplicates (n=3) and error bars indicate standard deviations from the mean. **(b)** Quantification of RNA products at early-/late-termination site. The efficiency at each terminator has been determined by the ratio of terminated species over terminated plus bypassed species. Different combinations of TFs are indicated below the plot. RNA was quantified in triplicates (n=3) and error bars indicate standard deviations from the mean. **(c)** Mathematical model used to simulate RNAP arriving at the early termination region over time and hypothetical scenarios to illustrate

the model: (I) All RNAPs terminate transcription ($P_{off} = 1$); (II) All RNAPs by-pass the early termination site ($P_{off} = 0$); (III, IV) Different fractions of RNAPs terminate and the rest by-passes (P_{off} = 0.8 and 0.3, respectively). **(d)** Parameter values (error estimates) from curve fitting to experimental triplicates with equation (3) (see Mathematical modelling for more details; A, amplitude – a coefficient reflecting the amount of paused RNAP; P_{off}, the probability for RNAP to terminate; τ_{pause}, the average pause time; σ, the width of the gaussian peak representing desynchronization of the RNAP population; t_0, average arrival time at terminator). A minimum value of 2 s was imposed for τ_{pause} (*). The limit was reached only for RNAP + Rho + NusG and RNAP + Rho + NusA + NusG (thus no error estimate could be made – indicated by star in parenthesis). **(e)** The negative control (NGN) shows that deletion of NusG-KOW abolishes the effect of NusG on Rho-dependent termination. The transcription profile looks similar to a reaction with RNAP in presence of Rho and NusA (compare to Fig. 4d). **(f)** The negative control (without Rho) shows no significant differences except NusA may stimulate slightly more pausing in the early termination region. All the source data are provided as a Source Data file.

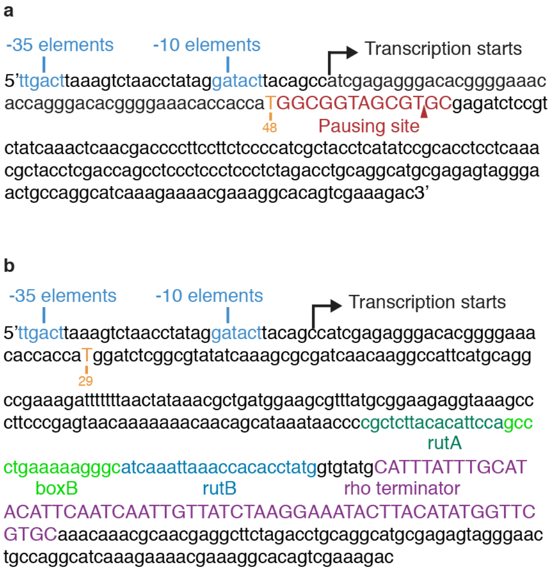

Supplementary Figure 7 DNA sequence with more detailed information. **(a)** DNA templates used in *ops* pause assays. T7A1 promotor contains the -35 and -10 elements, coloured in blue. The A48 position is coloured in orange; the ops pause is coloured in red. The triangle marked the pausing position. The black arrow indicated the initiation of the transcription. **(b)** Template DNA used for the *rho*-dependent termination assay. The -35 and -10 elements from the T7A1 promotor are coloured in blue; the A48 position is coloured in orange. the rutA, boxB, rutB are coloured in dark green, light green, dark blue and purple, respectively. The black arrow indicated the initiation of the transcription.

Table 1

Module	Subunit	Residues
Core	α1 α2<br>β<br>β′	all<br>1-26, 514-828, 1060-1235<br>504-771
Shelf	β<br>β′<br>ω	1236-1309<br>337-501;805-1323; 1356-1407<br>all
Clamp	β<br>β′	1318-1342<br>1-336,1324-1355
Swivel module	β<br>β′<br>ω	1236-1342<br>1-503, 805-931, 1127-1407<br>all
β-Lobe	β	143-448
β-Flap	β	829-937, 1040-1059
β-Flap-tip	β	891-912
β-Flap-tip-helix	β	897-907
β-protrusion	β	31-150, 456-512
β-SI1	β	227-340
β-SI2	β	938-1039
β′-SI3	β′	945-1130
Bridge helix (BH)	β′	770-804
β-gate loop	β	368-376

Table 2

Comparison of swivel module rotations for selected structures (done in PyMOL)

**Complex from present study**	**Rotation angle (6RH3)**	**Rotation angle (6ALH)**	**PDB ID**	**Reference**
EC non-swivelled (class1)	1.36°	~0° *	7Q0K	present study
EC swivelled (class2)	3.31°	2.1°	7Q0J	present study
NusG consensus	2.46°	1.12°	7PY1	present study
NusG non-swivelled (class 3)	1.97°	0.6°	7PY8	present study
NusG swivelled (class 5)	4.38°	3.0°	7PY0	present study
NusA consensus	3.29°	2.1°	7PY3	present study
NusA non-swivelled	1.26°	~0° *	7PYJ	present study
NusA swivelled	5.36°	4.17°	7PYK	present study
NusA NusG consensus	2.58°	1.24°	7PY5	present study
NusA NusG non-swivelled	1.63°	~0° *	7PY6	present study
NusA NusG swivelled	4.5°	3.28°	7PY7	present study
**Complex**	**Rotation angle**		**PDB ID**	
substrate bound (reactivated) RNAP	reference	-1.49°	6RH3	[23]
Q21-antitermination (loading complex)	~0° *	-1.37°	6P18	[33]
Q21-antitermination (loaded complex)	1.27°	~0° *	6P19	[33]
RNAP-EC	1.5°	reference	6ALH	[35]
NusG-opsEC	2.65°	1.29°	6C6U	[10]
RfaH-opsEC	2.59°	1.36°	6C6S	[10]
NusA-hisPEC	5.84°	4.66°	6FLQ	[7]
hisPEC	4.63°	3.57°	6ASX	[21]
Rho-dependent pre-termination complex (NusG bound)	4.55°	2.97°	6XAV	[12]
Rho-dependent pre-termination complex	4.98°	3.37°	6XAS	[12]
Transcription termination intermediate complex 1**	3.65°	2.25°	6Z9P	[11]
Transcription termination intermediate complex 2**	4.33°	3.14°	6Z9Q	[11]
Transcription termination intermediate complex 3**	4.55°	3.25°	6Z9R	[11]

***At very similar swivel module orientations, the rotation angle measurements are not meaningful because the orientation of the rotation axis cannot be reliably determined**

****Please note that we only compared swivel module orientations for RNAP with the clamp domain in a closed conformation**

Table 3. Refinement and model statistics for NusG-EC

Data collection	NusG-EC All particles	NusG-EC Class1 (no NusG & non swivelled)	NusG-EC Class2 (no NusG & swivelled)	NusG-EC Class3 (non swivelled)	NusG-EC Class4 (mid swivelled)	NusG-EC Class5 (more swivelled)	NusG-EC Consensus (Class3-class5)
Particles	367944	78470	67666	92561	66188	63059	221844
Pixel size (Å)	1.1						
Defocus range (um)	-0.8 to -3 µm						
Voltage (kV)	300						
Electron dose (e^{-} Å^{-2})	~ 50 e-/Å^2						
**PDB ID**	**-**	**7Q0K**	**7Q0J**	**7PY8**		**7PY0**	**7PY1**
**Model composition**							
Non-hydrogen atoms	27427	26439	26056	27443	27427	27443	27443
Protein residues	3307	3184	3190	3307	3307	3307	3307
RNA bases	11	11	11	11	11	11	11
DNA bases	64	64	64	64	64	64	64
Ligands (Zn^{2+}/Mg^{2+})	2/1	2/1	2/1	2/1	2/1	2/1	2/1
**Refinement**	**Consensus Refinement**	**Refinements of Classes**					
Nominal resolution (Å)	3.6	4	4.3	3.9	4.4	4.5	3.75
Map sharpening B-factor (Å^2)	156.0	134.4	155.1	137.6	168.5	149.9	154.0
Map cross-correlation (within mask)	0.84	0.82	0.85	0.82	0.85	0.85	0.76
Average B factor (Å^2)	124.4	132.82	213.96	138.69	230.10	235.61	96.61
**RMS deviations**							
Bond lengths (Å)	0.01	0.003	0.004	0.002	0.008	0.003	0.002
Bond angles (°)	0.933	0.642	0.791	0.523	1.339	0.699	0.564
**Ramachandran plot**							
Favored (%)	87.55	95.01	93.25	95.62	91.45	95.43	95.16
Allowed (%)	12.45	4.99	6.65	4.38	8.37	4.57	4.78
Outliers (%)	0.0	0.00	0.09	0.00	0.18	0.00	0.06
**Validation**							
Molprobity Score	2.3	1.93	2.02	1.74	2.45	1.82	1.77
Molprobity Clash score	15.18	11.58	11.65	7.91	28.85	9.45	7.87
Rotamer outliers (%)	0.07	0.00	0.04	0.04	0.0	0.04	0.04

Table 4. Refinement and model statistics for NusA-EC

**Data collection**	**NusA-EC Consensus**	**NusA-EC swivelled**	**NusA-EC non-swivelled**
Particles	161665	43495	36328
Pixel size (Å)<br>Defocus range (um)<br>Voltage (kV)<br>Electron dose (e^{-} $Å^{-2}$)	1.09<br>-0.8 to -3 µm<br>300<br>~ 50 e-/Å2		
**PDB ID**	**7PY3**	**7PYK**	**7PYJ**
**Model composition**			
Non-hydrogen atoms	29362	29362	29362
Protein residues	3787	3787	3787
RNA bases	11	11	11
DNA bases	57	57	57
Ligands (Zn^{2+}/Mg^{2+})	2/1	2/1	2/1
**Refinement**	**Consensus Refinement**	**Refinements of Classes**	
Nominal resolution (Å)	3.77	4.1	4.2
Map sharpening B-factor ($Å^2$)	142.3	136.7	133.1
Map cross-correlation (within mask)	0.78	0.85	0.85
Average B factor ($Å^2$)	154.02	253.96	281.34
**RMS deviations**			
Bond lengths (Å)	0.003	0.004	0.003
Bond angles (°)	0.678	0.773	0.681
**Ramachandran plot**			
Favored (%)	93.58	93.34	93.28
Allowed (%)	6.42	6.66	6.66
Outliers (%)	0.00	0.00	0.05
**Validation**			
Molprobity Score	1.87	2.03	2.02
Molprobity Clash score	8.26	12.23	11.81
Rotamer outliers (%)	0.04	0.04	0.04

Table 5. Refinement and model statistics for NusA-NusG-EC

**Data collection**	**NusA-NusG-EC All particles**	**NusA-NusG-consensus**	**Class NusG EC**	**NusA-NusG-EC Non-swivelled**	**NusA-NusG-EC swivelled**
Particles	409804	259835	149969	94643	89013
Pixel size (Å)<br>Defocus range (um)<br>Voltage (kV)<br>Electron dose (e^{-} Å^{-2})	1.1<br>-0.8 to -3 µm<br>300<br>~ 50 e-/Å2				
**PDB ID**	**-**	**7PY5**	**-**	**7PY6**	**7PY7**
**Model composition**					
Non-hydrogen atoms	29936	29960	29936	29960	30001
Protein residues	3832	3832	3832	3832	3832
RNA bases	11	11	11	11	11
DNA bases	56	56	56	54	56
Ligands (Zn^{2+}/Mg^{2+})	2/1	2/1	2/1	2/1	2/1
**Refinement**	**Consensus Refinement**	**Refinements of Classes**			
Nominal resolution (Å)	3.9	3.86	4.1	4.1	4.2
Map sharpening B-factor (Å^2)	171.8	151.7	177.0	146.2	152.7
Map cross-correlation (within mask)	0.84	0.79	0.75	0.83	0.83
Average B factor (Å^2)	107.97	76.46	?	139.79	202.12
**RMS deviations**					
Bond lengths (Å)	0.009	0.002	0.004	0.003	0.004
Bond angles (°)	1.254	0.623	0.723	0.652	0.666
**Ramachandran plot**					
Favored (%)	89.87	94.6	89.87	95.51	94.23
Allowed (%)	9.94	5.4	9.94	4.49	5.69
Outliers (%)	0.18	0.0	0.18	0.00	0.08
**Validation**					
Molprobity Score	2.33	1.83	2.33	1.84	1.94
Molprobity Clash score	19	8.59	19	10.18	10.67
Rotamer outliers (%)	0.0	0.00	0.0	0.04	0.04
