# Supplementary material for: Transcription factors modulate RNA polymerase conformational equilibrium
Source: Nat Commun. 2022 Mar 22;13:1546. doi: 10.1038/s41467-022-29148-0 (PMC8940904; doi:10.1038/s41467-022-29148-0)
Supplement: Supplementary file 2 — Description of additional Supplementary File [file 41467_2022_29148_MOESM2_ESM.pdf]

<u>Descriptions of additional Supplementary Files</u>

Supplementary Movie S1: Swivelling of the NusG-EC. Morphing between NusG-EC class 3 (light-green) and class 5 (purple) shows how NusG (red) moves with the swivel module. The β-protrusion (dark blue) is flexible and can accommodate NusG in a more swivelled conformation.

Supplementary Movie S2: Swivelling of the NusA-EC. The movie shows a morph based on 3D-variability analysis performed in cryoSPARC between a non-swivelled (orange) and swivelled (purple) NusA-EC at low contour level. Cartoon models illustrate how NusA (yellow) can approach the RNAP β''-ZF in the swivelled conformation. Additional density for the nascent transcript is indicated (arrow). In the nonswivelled conformation, NusA approaches the **ω**-domain of RNAP.
