# Supplementary material for: Transcription factors modulate RNA polymerase conformational equilibrium
Source: Nat Commun. 2022 Mar 22;13:1546. doi: 10.1038/s41467-022-29148-0 (PMC8940904; doi:10.1038/s41467-022-29148-0)
Supplement: Supplementary file 5 — Reporting Summary [file 41467_2022_29148_MOESM5_ESM.pdf]

nature portfolio

Corresponding author(s): Albert Weixlbaumer

Last updated by author(s): Dec 14, 2021

Reporting Summary

Nature Portfolio wishes to improve the reproducibility of the work that we publish. This form provides structure for consistency and transparency in reporting. For further information on Nature Portfolio policies, see our Editorial Policies and the Editorial Policy Checklist.

Statistics

For all statistical analyses, confirm that the following items are present in the figure legend, table legend, main text, or Methods section.

n/a	Confirmed	
☐	☒	The exact sample size (*n*) for each experimental group/condition, given as a discrete number and unit of measurement
☐	☒	A statement on whether measurements were taken from distinct samples or whether the same sample was measured repeatedly
☒	☐	The statistical test(s) used AND whether they are one- or two-sided<br>*Only common tests should be described solely by name; describe more complex techniques in the Methods section.*
☒	☐	A description of all covariates tested
☒	☐	A description of any assumptions or corrections, such as tests of normality and adjustment for multiple comparisons
☐	☒	A full description of the statistical parameters including central tendency (e.g. means) or other basic estimates (e.g. regression coefficient) AND variation (e.g. standard deviation) or associated estimates of uncertainty (e.g. confidence intervals)
☒	☐	For null hypothesis testing, the test statistic (e.g. *F*, *t*, *r*) with confidence intervals, effect sizes, degrees of freedom and *P* value noted<br>*Give P values as exact values whenever suitable.*
☒	☐	For Bayesian analysis, information on the choice of priors and Markov chain Monte Carlo settings
☒	☐	For hierarchical and complex designs, identification of the appropriate level for tests and full reporting of outcomes
☒	☐	Estimates of effect sizes (e.g. Cohen's *d*, Pearson's *r*), indicating how they were calculated

Our web collection on statistics for biologists contains articles on many of the points above.

Software and code

Policy information about availability of computer code

Data collection	SerialEM ver. 3.6 was used for cryo-EM data collection (http://bio3d.colorado.edu/SerialEM/).
Data analysis	Software used for data analysis in this study are available online:<br>1. MotionCor2 (http://msg.ucsf.edu/em/software/motioncor2.html): particle motion correction<br>2. Gctf 1.06 (http://www.mrc-lmb.cam.ac.uk/kzhang/Gctf/): CTF estimation<br>3. CTFFIND4 (https://grigoriefflab.umassmed.edu/ctffind4): CTF estimation<br>4. EMAN2 (https://blake.bcm.edu/emanwiki/EMAN2): Cryo-EM data analysis<br>5. RELION 3.1 (http://www2.mrc-lmb.cam.ac.uk/relion): Cryo-EM data analysis<br>6. cryoSPARC 3.1 (https://cryosparc.com): Cryo-EM data analysis<br>7. Coot 9.0 (https://www2.mrc-lmb.cam.ac.uk/ personal/pemsley/coot/): Model building<br>8. UCSF Chimera 1.14 (https://www.cgl.ucsf.edu/chimera/): Density map and structural model visualization<br>9. UCSF ChimeraX 1.1 (https://www.rbvi.ucsf.edu/chimerax/): Structural figure preparation<br>10. PyMOL 1.6 (https://www.pymol.org/): Structural figure preparation<br>11. Phenix 1.19 (https://www.phenix-online.org): Model refinement<br>12. DeepEMhancer 0.13 (https://github.com/rsanchezgarc/deepEMhancer): Map sharpening

For manuscripts utilizing custom algorithms or software that are central to the research but not yet described in published literature, software must be made available to editors and reviewers. We strongly encourage code deposition in a community repository (e.g. GitHub). See the Nature Portfolio guidelines for submitting code & software for further information.

Data

Policy information about availability of data

All manuscripts must include a data availability statement. This statement should provide the following information, where applicable:

- Accession codes, unique identifiers, or web links for publicly available datasets
- A description of any restrictions on data availability
- For clinical datasets or third party data, please ensure that the statement adheres to our policy

Model coordinates and density maps will be availbe in the Protein Data Bank and EM Data Bank.
The accession numbers for the eleven cryo-EM reconstructions (RNAP-EC non-swivelled, RNAP-EC swivelled, NusG-EC consensus, NusG-EC non-swivelled, NusG-EC swivelled, NusA-EC consensus, NusA-EC non-swivelled, NusA-EC swivelled, NusA-NusG-EC consensus, NusA-NusG-EC non-swivelled, NusA-NusG-EC swivelled,) reported in this paper are EMD-13746, EMD-13745, EMD-13707, EMD-13716, EMD-13706, EMD-13709, EMD-13717, EMD-13718, EMD-13713, EMD-13714, and EMD-13715, respectively. Fitted models were deposited in the RCSB Protein Data Bank (https://www.rcsb.org/) with accession codes 7Q0K (RNAP-EC non-swivelled), 7Q0J (RNAP-EC swivelled), 7PY1 (NusG-EC consensus), 7PY8 (NusG-EC non- swivelled), 7PY0 (NusG-EC swivelled), 7PY3 (NusA-EC consensus), 7PYJ (NusA-EC non- swivelled), 7PYK (NusA-EC swivelled), 7PY5 (NusA-NusG-EC consensus), 7PY6 (NusA-NusG-EC non- swivelled), and 7PY7 (NusA-NusG-EC swivelled).

Field-specific reporting

Please select the one below that is the best fit for your research. If you are not sure, read the appropriate sections before making your selection.

- [x] Life sciences
- [ ] Behavioural & social sciences
- [ ] Ecological, evolutionary & environmental sciences

For a reference copy of the document with all sections, see nature.com/documents/nr-reporting-summary-flat.pdf

Life sciences study design

All studies must disclose on these points even when the disclosure is negative.

Sample size	Sample size (particle numbers) in Cryo-EM datasets was the result of available time on high-end microscopes and whether or not increasing the number of particles would result in higher resolution or not.<br>Sample size in biochemical experiments was chosen based on practical considerations and experiments were done in at least triplicates.
Data exclusions	Some Cryo-EM data was excluded after 2D and 3D classification. This follows standard procedures, uses cross-correlation as the metric and is carried out in an objective way by data processing software (particle classification in relion and heterogeneous refinement procedures in CryoSPARC).
Replication	All attempts at replication were successful. Structural heterogeneity results were reproducible and two independent programs (relion and CryoSPARC) gave consistent results. Replication of biochemical experiments were also successful.<br>Biochemical experiments were done in at least triplicates.
Randomization	Randomization is not relevant to this study. Cryo-EM particles are assigned to structural states by software programs, which us cross-correlation and follows standard procedures. Each biochemical experiment was done in triplicates and is its own experimental group with no need to assign it to a specific one.
Blinding	Not applicable to this study

Reporting for specific materials, systems and methods

We require information from authors about some types of materials, experimental systems and methods used in many studies. Here, indicate whether each material, system or method listed is relevant to your study. If you are not sure if a list item applies to your research, read the appropriate section before selecting a response.

Materials & experimental systems

n/a	Involved in the study
x	Antibodies
x	Eukaryotic cell lines
x	Palaeontology and archaeology
x	Animals and other organisms
x	Human research participants
x	Clinical data
x	Dual use research of concern

Methods

n/a	Involved in the study
x	ChIP-seq
x	Flow cytometry
x	MRI-based neuroimaging
